# Management of Acute Coronary Syndromes in Older People: Comprehensive Review and Multidisciplinary Practice-Based Recommendations

**DOI:** 10.3390/jcm13154416

**Published:** 2024-07-28

**Authors:** Ahthavan Narendren, Natalie Whitehead, Louise M. Burrell, Matias B. Yudi, Julian Yeoh, Nicholas Jones, Laurence Weinberg, Lachlan F. Miles, Han S. Lim, David J. Clark, Ali Al-Fiadh, Omar Farouque, Anoop N. Koshy

**Affiliations:** 1 Department of Cardiology, Austin Health, Heidelberg, VIC 3084, Australia; ahthavan6@gmail.com (A.N.); natalie.whitehead16@hotmail.com (N.W.); l.burrell@unimelb.edu.au (L.M.B.); matiasyudi@gmail.com (M.B.Y.); julian.yeoh@austin.org.au (J.Y.); nicholas.jones@austin.org.au (N.J.); lim.h@unimelb.edu.au (H.S.L.); clarkdavidj@hotmail.com (D.J.C.); ali.al-fiadh@austin.org.au (A.A.-F.); omar.farouque@austin.org.au (O.F.); 2 Department of Cardiology, Northern Health, Epping, VIC 3076, Australia; 3 Department of Medicine, The University of Melbourne, Melbourne, VIC 3052, Australia; 4 Department of Critical Care, The University of Melbourne, Melbourne, VIC 3010, Australia; laurence.weinberg@austin.org.au (L.W.); lachlan.miles@austin.org.au (L.F.M.); 5 Department of Anaesthesia, Austin Health, Heidelberg, VIC 3084, Australia; 6 Department of Cardiology, The Royal Melbourne Hospital, Parkville, VIC 3052, Australia

**Keywords:** acute coronary syndrome, coronary artery disease, cardiovascular disease, older adults, octogenarians, multimorbidity, frailty

## Abstract

Managing health care for older adults aged 75 years and older can pose unique challenges stemming from age-related physiological differences and comorbidities, along with elevated risk of delirium, frailty, disability, and polypharmacy. This review is aimed at providing a comprehensive analysis of the management of acute coronary syndromes (ACS) in older patients, a demographic substantially underrepresented in major clinical trials. Because older patients often exhibit atypical ACS symptoms, a nuanced diagnostic and risk stratification approach is necessary. We aim to address diagnostic challenges for older populations and highlight the diminished sensitivity of traditional symptoms with age, and the importance of biomarkers and imaging techniques tailored for older patients. Additionally, we review the efficacy and safety of pharmacological agents for ACS management in older people, emphasizing the need for a personalized and shared decision-making approach to treatment. This review also explores revascularization strategies, considering the implications of invasive procedures in older people, and weighing the potential benefits against the heightened procedural risks, particularly with surgical revascularization techniques. We explore the perioperative management of older patients experiencing myocardial infarction in the setting of noncardiac surgeries, including preoperative risk stratification and postoperative care considerations. Furthermore, we highlight the critical role of a multidisciplinary approach involving cardiologists, geriatricians, general and internal medicine physicians, primary care physicians, and allied health, to ensure a holistic care pathway in this patient cohort.

## 1. Introduction

**Figure 1:**
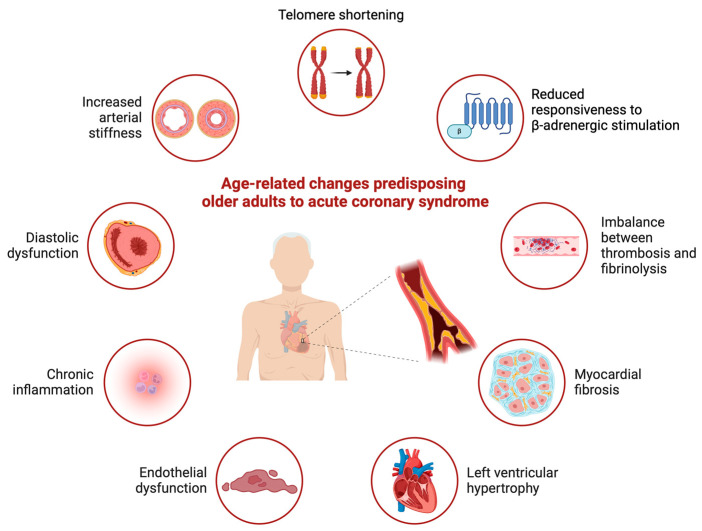
Age-related changes predisposing older adults to acute coronary syndromes.

By 2050, approximately 16% of the global population will be ≥65 years of age, with a projected life expectancy of 77.2 years [1]. Although the term “older adult” has been used to refer to a range of age subgroups in the medical literature, the American College of Cardiology/American Heart Association’s Guideline for the Evaluation and Diagnosis of Chest Pain defines older adults as those ≥ 75 years of age [2]. Approximately 35–40% of all cases of acute coronary syndrome (ACS) occur in older adults, who have an 8-year mortality rate of 77% after myocardial infarction (MI) [3,4,5,6]. Older adults exhibit age-related changes that predispose them to ACS (Figure 1), including an increased prevalence of inflammaging [7]. This phenomenon, characterized by a chronic pro-inflammatory state, is a major contributor to the development and progression of atherosclerosis. Nonetheless, older adults remain substantially underrepresented in major clinical trials, thus leading to a paucity of evidence-based guidelines tailored to this population [8]. Consequently, the management of older patients with ACS is often guided by extrapolated trial findings from younger populations.

Older patients are considerably less likely to be treated with optimal guideline-directed medical therapy and more than twice as likely to receive conservative medical management, than younger patients [5,9]. Older patients undergoing conservative medical management for non-ST-segment elevation MI (NSTEMI) have nearly two-fold greater mortality than those treated with percutaneous coronary intervention (PCI) or coronary artery bypass grafting (CABG) [9]. Regardless of the selected treatment approach, older patients with ACS have significantly higher in-hospital mortality rates than younger patients.

A notable gap exists in cardiology guidelines regarding the diagnosis and management of ACS in older adults. These patients can pose diagnostic and management challenges, often because of age-related physiological differences and the presence of concurrent geriatric syndromes (Figure 1). Addressing ACS in the context of these geriatric comorbidities, with the inclusion of a multidisciplinary team (MDT), is essential for providing effective treatment to this patient cohort, where competing life-limiting conditions often exist. This review emphasizes the need for using a tailored and individualized approach to ACS diagnosis and management in older adults while considering the intricate interplay of comorbidities and the unique challenges associated with aging.

## 2. ACS Evaluation

The term ACS encompasses a group of conditions including ST-segment elevation MI (STEMI), NSTEMI, and unstable angina, which involve acute myocardial ischemia or infarction [10].

**Figure 2:**
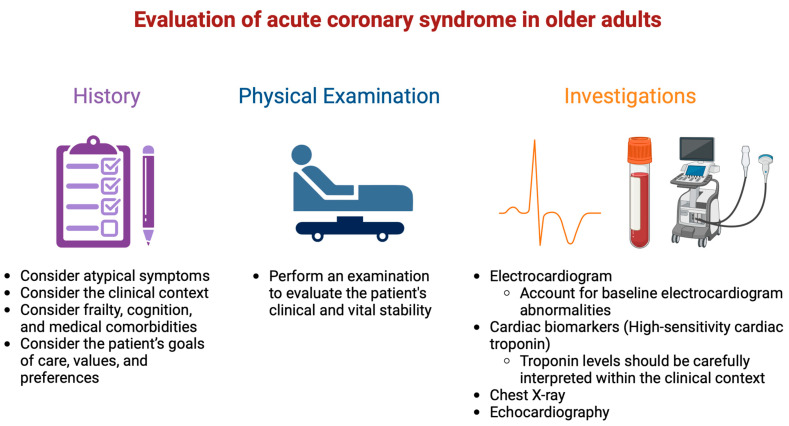
Evaluation of acute coronary syndrome in older adults.

Myocardial ischemia results from an imbalance between oxygen supply and demand and can be identified from the patient’s history and ECG [10]. Chest pain is a common presenting symptom in patients with ACS. All patients presenting with chest pain, regardless of age, should be promptly assessed to exclude life-threatening conditions such as ACS, pulmonary embolism, aortic dissection, esophageal rupture, or tension pneumothorax [2]. A fundamental component of this evaluation is an initial electrocardiogram (ECG), supported by a physical examination, cardiac biomarkers, and most importantly a comprehensive history (Figure 2). 

Older patients, however, are more likely than younger patients to exhibit atypical symptoms, thus further complicating the diagnostic workup of ACS [11]. Among older adults presenting with MI, 44% do not report chest pain as their primary symptom, including 40% of patients with STEMI [12]. Additionally, in patients presenting with NSTEMI, women are less likely than men to present with chest pain as their primary symptom. A total of 13% of patients ≥ 75 years of age hospitalized with acute MI present with respiratory symptoms as their primary complaint, 11% present with any other discomfort, 6% present with gastrointestinal symptoms, 3% present with fatigue or weakness, and 3% are asymptomatic at presentation. Several possible mechanisms may contribute to the presentation of atypical chest pain in older patients, including reduced pain perception due to increased pain thresholds to short-duration noxious stimuli and an increased prevalence of comorbidities such as diabetes that can lead to cardiac autonomic dysfunction [13,14]. Cardiac autonomic dysfunction is significantly associated with a higher risk of silent myocardial ischemic events [15]. Delays in recognizing ACS in older patients who are asymptomatic or present with atypical symptoms may be associated with delayed treatment and increased long-term mortality [16].

### 2.1. ECG Interpretation and ACS Diagnosis in Older Adults

ECG interpretation in older adults can be challenging because as many as 70% of adults ≥ 75 years of age exhibit baseline ECG abnormalities [17]. Approximately 9% of older adults have atrial fibrillation (AF), 13% have first-degree atrioventricular block, 10% have right bundle branch block, and 20% show signs of left ventricular hypertrophy. These abnormalities lead to a 50% higher rate of false positive ACS diagnoses [18]. Although these abnormalities can indicate a higher risk of future adverse cardiovascular events, their prevalence limits the effectiveness of ECG screening in diagnosing ACS in older patients.

High-sensitivity cardiac troponin T/I (hs-cTn T/I) is widely used in the diagnostic workup of patients with ACS but has lower diagnostic accuracy in older patients relative to younger patients [19]. Routine troponin testing is not recommended for older people with non-specific symptoms. In a study of 594 patients ≥ 65 years of age presenting with nonspecific symptoms, 69% underwent evaluation with troponin testing, 20% of whom had elevated troponin levels [20]. However, only 1.2% of patients tested for troponin levels received a diagnosis of ACS. Elevated troponin levels are often associated with conditions such as chronic obstructive pulmonary disease, cardiomyopathies, diabetes, anemia, and renal insufficiency—comorbidities commonly encountered in older people. Among patients ≥ 65 years of age, 73% have elevated cardiac troponin T without a clinical diagnosis of ACS, thus highlighting the need to reassess conventional cut-off values in this population and adopt careful interpretation [21]. Cardiac myosin-binding protein C (cMyC) is a novel cardiac biomarker with the potential to aid in diagnosing and predicting cardiovascular events [22]. Its interpretation should be considered alongside other markers, such as cardiac troponins. Although no statistically significant differences in cMyC concentrations have been observed with age, further research is required to analyze the prognostic utility of cMyC in older adults.

Myocardial injury is characterized by an elevated cardiac troponin level above the 99th percentile and is classified as acute if a significant rise and/or fall in levels is observed [10]. Type 1 MI (T1MI) is due to atherothrombotic coronary artery disease (CAD) and is typically precipitated by rupture or erosion of atherosclerotic plaques. In contrast, type 2 MI (T2MI) is characterized by ischemic myocardial injury resulting from an imbalance between myocardial oxygen supply and demand, without plaque disruption. Among patients with elevated troponin, the incidence of T2MI is approximately 21%, while T1MI comprises 46%, and myocardial injury comprises 33% [23]. The median age of patients with T2MI is 81 years, and that of myocardial injury is 84 years. In contrast, the median age for patients with T1MI is 67 years. Patients with T2MI are likely to be older and have more comorbidities [24]. The mechanisms underlying T2MI are due to age-associated physiological cardiovascular decline; predisposing factors such as chronic anemia and valvular disease such as severe aortic stenosis; and acute triggering factors such as acute infections, tachyarrhythmias, and acute decompensated heart failure [25]. Given the extensive list of differential diagnoses associated with elevated troponin levels in older patients, a thorough diagnostic evaluation should be conducted as treatment approaches can vary widely according to the underlying cause of troponin elevation. 

### 2.2. Use of Non-Invasive Imaging Modalities

Cardiovascular imaging is often used in the diagnostic workup of ACS. However, various confounding factors that might influence test results in older patients must be considered. Cognitive impairment can impede patients’ ability to follow instructions, whereas conditions such as kyphoscoliosis, arthritis, and changes in body habitus can make positioning patients for optimal imaging challenging, reducing its diagnostic accuracy [26].

Bedside echocardiography is a valuable tool for the diagnosis of ACS in older patients, particularly in the setting of a rise in troponin coupled with atypical symptoms or baseline ECG abnormalities, which are frequently observed in older people [27]. Bedside echocardiography enables the detection of regional wall motion abnormalities suggestive of CAD and mechanical complications of MI, including mitral regurgitation, pericardial effusion, LV thrombus, free wall rupture, and ventricular septal rupture [28]. Bedside echocardiography can also aid in detecting severe valvular disease before cardiac catheterization and thus guide management decisions [27]. Additionally, echocardiography is instrumental in the diagnosis of Takotsubo cardiomyopathy, which is most commonly found in older women. The pathophysiology of Takotsubo syndrome is complex, reflecting the systemic physiological response to acute, severe stress [29]. This response leads to persistent myocardial stunning, causing diffuse regional wall motion abnormalities despite the absence of significant obstructive CAD, with spontaneous recovery of myocardial function typically occurring within days or weeks [30].

Coronary computed tomography angiography (CCTA) is a non-invasive imaging modality capable of quantifying atherosclerotic plaque burden, as well as diagnosing the extent and severity of obstructive and non-obstructive CAD, without the challenges associated with exercise or pharmacological stress, which are common in older adults. CCTA is recommended as a class 1, level 1A recommendation to exclude atherosclerotic plaques and obstructive CAD in patients at intermediate risk of ACS who present with acute chest pain and no history of CAD, following an inconclusive or negative evaluation for ACS [2]. Whereas clinicians may use CCTA for triage of younger patients with suspected ACS through assessment of high-risk plaques, an age-specific approach is crucial when considering the use of CCTA in older populations. 

Several limitations of CCTA should be considered when this diagnostic modality is used for the evaluation of older patients with suspected ACS. Densely calcified plaques, which are more common in older patients, can impair the accuracy and quality of CCTA interpretation because of blooming artifacts and can lead to overestimation of luminal stenosis [31]. CCTA also requires intravenous contrast, which may pose limitations in patients with underlying renal impairment, and tachycardia can also preclude the acquisition of high-quality images [26]. Although CCTA is safer and less expensive than invasive coronary angiography, the limitations of CCTA should be considered, given its lower diagnostic accuracy in older adults. 

## 3. Pharmacological Management

Older patients are less likely than younger patients to receive guideline-directed medical therapy in the management of ACS, although age is an independent risk factor for cardiovascular disease burden and mortality [32,33]. Several factors influence this treatment–risk paradox. Aging can affect both pharmacokinetics and pharmacodynamics, by decreasing kidney function and hepatic perfusion, causing changes in body fat and muscle mass composition, and potentially influencing drug tolerability and the risk of drug-related adverse effects [33,34]. Trials of pharmacotherapy in ACS often exclude or underrepresent patients > 75 years of age [35,36]. Older patients frequently have multiple comorbidities with many drug–disease interactions (Table 1). Finally, polypharmacy (the use of five or more medications) is common in older populations, thus increasing the risk of drug–drug interactions (Table 1). The addition of further medications after ACS may increase the risk of medication errors, lack of adherence, and hospitalization [37]. Dexterity and visual impairment may also impair the ability of an older adult to comply with the recommended pharmacotherapy [38]. While dose administration aids have helped in this aspect, they can also add to the cost of treatment. Pharmacological management of ACS in this context, as well as its potential to influence the risk of falls, confusion and cognition, frailty, and function, must be carefully considered, as does the evidence base with regard to treatment recommendations [39]. 

*Recommendation: When prescribing pharmacological therapy in older adults post-ACS, consideration should be given to minimizing medication-related risks. This can be achieved by involving pharmacy services early during hospital admission and at discharge, with home visits, simplifying medication regimens with combination tablets or once-daily dosing where possible, deprescribing when appropriate, and rationalizing medications to reduce polypharmacy.*

### 3.1. Dual Antiplatelet Therapy

**Figure 3:**
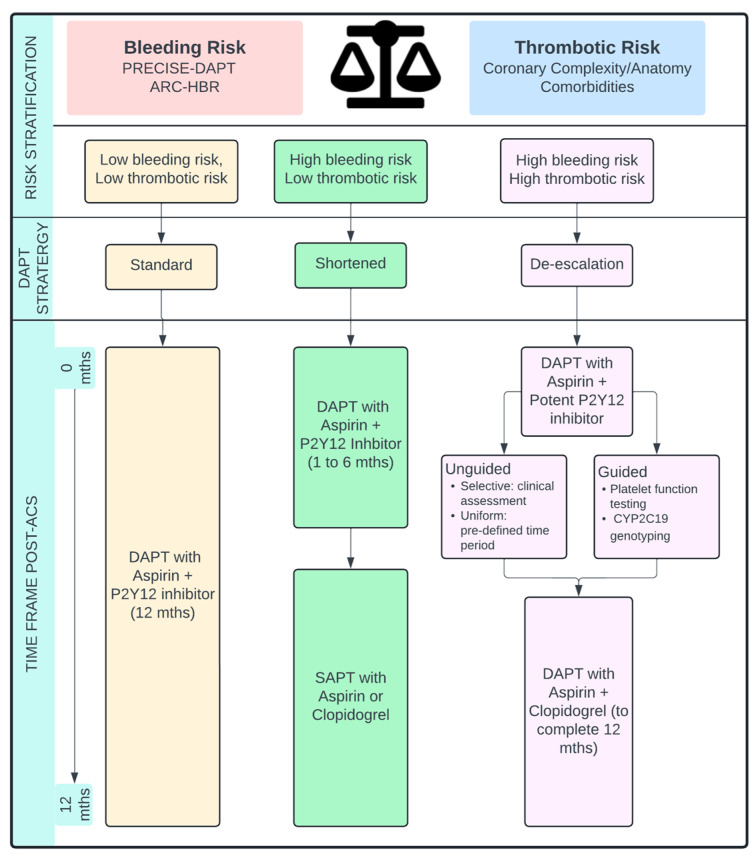
Proposed risk stratification guide for antiplatelet strategies in older patients post-acute coronary syndrome. ARC-HBR: Academic Research Consortium for High Bleeding Risk; DAPT: dual antiplatelet therapy; mths: months; SAPT: single antiplatelet platelet therapy.

Low-dose aspirin in combination with a potent P2Y12 inhibitor (prasugrel or ticagrelor) for 12 months after ACS is the recommended standard dual antiplatelet therapy (DAPT) strategy, which is superior to clopidogrel in decreasing ischemic events, despite posing a higher bleeding risk [33,34]. However, DAPT therapy in older populations after ACS can be challenging, because multiple comorbidities as well as complex coronary disease are often present, thus increasing bleeding and/or thrombotic risk. The 2021 American College of Cardiology/American Heart Association Guideline for Coronary Artery Revascularization, suggests that more potent P2Y12 inhibitors should be used with caution in older adults receiving standard DAPT therapy due to increased bleeding risk [36]. Similarly, the recent 2023 European Society of Cardiology Guideline for the Management of Acute Coronary Syndromes, has newly recommended the consideration of clopidogrel as the P2Y12 inhibitor in older patients with ACS (≥ 70 years of age), particularly in those with a high bleeding risk (class 2B, level B recommendation) [35]. Consideration should therefore be given to bleeding risk versus thrombotic risk, as well as standard versus alternative DAPT strategies, to better individualize DAPT therapy in older patients (Figure 3).

#### 3.1.1. Assessing Bleeding and Thrombotic Risk 

Two clinical scores are recommended for evaluating older patients at high bleeding risk (HBR) receiving DAPT therapy after ACS [40]. The PRECISE-DAPT score includes five criteria (age, creatinine clearance, hemoglobin level, white blood cell count, and previous spontaneous bleeding) to predict the risk of out-of-hospital bleeding with DAPT, with a score ≥ 25 indicating HBR [41]. The Academic Research Consortium for High Bleeding Risk (ARC-HBR) developed a consensus definition of clinical and biochemical data wherein patients are considered to have HBR if at least one major and two minor criteria are present (Table 2) [42]. Given that age is a criterion in both scoring methods, older patients are frequently identified as having HBR. High thrombotic risk can be considered in patients with previous stent thrombosis while receiving antiplatelet treatment, and complex PCI (three vessels treated, three or more stents implanted, three or more lesions treated, bifurcation with two stents implanted, total stent length > 60 mm, surgical bypass graft or chronic total occlusion at target lesions, atherectomy device use, or left main PCI) [43]. Consideration should also be paid to ischemic risk and comorbidities contributing to cardiovascular risk factors. A post hoc analysis of the Elderly ACS II trial, comparing reduced dose prasugrel (5 mg daily) with clopidogrel, has identified that potent P2Y12 inhibition significantly decreases the risk of ischemic events in the 30 days after ACS but increases rates of bleeding in the following 31–365 days [44]. Thrombotic risk after ACS is high in the acute phase and gradually decreases over time, whereas the bleeding risk remains constant; therefore, DAPT therapy and potentially its duration can be tailored to better match this profile in older people.

#### 3.1.2. Standard DAPT Therapy

In critical trials of ticagrelor and prasugrel, older patients ≥ 75 years of age have been underrepresented. This population accounted for only 15.5% (*n* = 2878) of participants in the PLATO trial and 13% (*n* = 1770) of participants in the TRITON-TIMI 38 trial [45,46]. Subsequently, several studies and post hoc analyses were conducted to provide further evidence of standard DAPT therapy in older people.

#### 3.1.3. Ticagrelor vs. Clopidogrel

There has been inconsistent evidence of the ischemic benefit of ticagrelor over clopidogrel in the older population as standard DAPT therapy. A substudy of the PLATO trial in older patients ≥ 75 years of age compared with those < 75 years of age receiving ticagrelor continued to have a reduction in ischemic events without an increase in the rate of major bleeding [47]. Comparatively, The POPular AGE randomized controlled trial (RCT), did not show improved ischemic outcomes with ticagrelor over clopidogrel, in patients ≥ 70 years of age and was associated with significantly elevated rates of bleeding [48]. Premature discontinuation of ticagrelor occurred in 47% (*n* = 238) of patients in this study, thus potentially limiting the potential benefits of ticagrelor but also possibly reflecting real-life limitations in maintaining high potency antiplatelet therapy in older patients [48]. A recent observational analysis from the SWEDEHEART registry, in patients with ACS ≥ 80 years of age prescribed ticagrelor versus clopidogrel, reported that patients receiving ticagrelor had a higher risk of death and bleeding than those treated with clopidogrel [49].

#### 3.1.4. Prasugrel vs. Clopidogrel

Prasugrel at full dose (10 mg daily) as standard DAPT therapy compared to clopidogrel in the older cohort of the TRITON-TIMI 38 trial, identified no net clinical benefit, driven by a significantly elevated risk of major and fatal bleeding [46]. Consequently, the European Medicines Agency and US Food and Drug Administration do not recommend the general use of prasugrel in older patients ≥ 75 years of age, unless high risk, and if used a reduced dose of 5 mg daily is recommended [34,50]. Reduced dose prasugrel versus clopidogrel has been investigated in older patients in two ACS trials. The Elderly ACS II trial, with patients > 74 years of age with ACS undergoing early PCI, did not show significant ischemic benefit with low dose prasugrel but did identify a higher rate of significant bleeding [51]. In the TRIOLOGY ACS subgroup analysis, among older patients ≥ 75 years of age receiving medical management after ACS, decreased dose prasugrel, compared with clopidogrel, resulted in no difference in ischemic or bleeding outcomes [52]. Real-world studies in all-comers, as well as a population of patients with stable ischemic heart disease undergoing revascularization, have indicated comparable ischemic and bleeding outcomes after stratification by P2Y_12_ inhibitor use [53].

#### 3.1.5. Alternative DAPT Strategies

De-escalation of DAPT entails a switch from a more potent P2Y12 inhibitor (ticagrelor or prasugrel) to clopidogrel in the 12-month period after ACS [35]. De-escalation of DAPT therapy can be guided by platelet function testing or CYP2C19 genotyping or can be unguided, on the basis of clinical assessment (unguided selective) or a predefined time period (unguided uniform) [54]. The goal of de-escalation is to maximize ischemic protection after the index event while minimizing bleeding risk (Figure 3).

Shortened DAPT therapy (≤ 6 months) in older patients with HBR and transitioning to single agent antiplatelet therapy, either aspirin or clopidogrel, is another strategy that may be considered (Figure 3). Trials assessing short-term DAPT compared with standard DAPT therapy in patients with ACS have ranged in duration from 1 to 6 months [55,56,57]. 

A recently published meta-analysis by Fujisaki et al. has compared the efficacy and safety of various DAPT strategies in older people (*n* = 47,911) after ACS from 16 trials [58]. The strategies were divided into DAPT with clopidogrel, DAPT with potent P2Y12 inhibitors, uniform de-escalation, guided de-escalation, and short-term DAPT. The study has concluded that a uniform de-escalation strategy, compared with DAPT using potent P2Y12 inhibitors, is associated with improved net clinical benefits [58]. Short-term DAPT was the least effective strategy for decreasing ischemic outcomes. However, short-term DAPT, compared with potent P2Y12 inhibitors, significantly decreased major bleeding and may be a reasonable option for older patients at HBR, without a high thrombotic risk [58].

*Recommendation: DAPT therapy in older adults should be individualized according to bleeding risk and thrombotic risk. Consideration should be given to de-escalating DAPT therapy, particularly if HBR is associated with high thrombotic risk (Figure 3). The use of risk stratification tools in this decision-making is warranted, and further research into the utility of artificial intelligence-based models, such as machine-based learning methods, to further individualize decision-making is required [59].*

#### 3.1.6. Combined Anticoagulant and Antiplatelet Therapy

Because the incidence of AF and stroke risk increases with age, many older patients after ACS may also have concurrent indications for anticoagulation for stroke prevention in AF, thus further increasing bleeding risk [60]. In the WOEST Trial, comparing dual antithrombotic Therapy (DAT), clopidogrel, and warfarin, to triple antithrombotic therapy (TAT), clopidogrel, aspirin, and warfarin, DAT was associated with a significant reduction in bleeding, without an increase in the rate of thrombotic events [61]. Subsequent trials have compared TAT or DAT strategies with direct-acting oral anticoagulants (DOACs) versus warfarin at varying durations, with DOACs demonstrating lower bleeding risk, without an increase in thrombotic events [62,63,64,65].

*Recommendation: Based on current evidence, consideration should be paid to only short-duration TAT (≤1–4 weeks) in older patients after PCI with an indication for anticoagulation. Preference should be given to DOACs over vitamin K antagonists, and DAPT with clopidogrel and aspirin [33]. Transition to DAT with a DOAC and clopidogrel should be continued for 6–12 months, and transition to DOAC alone should occur thereafter unless a significantly high ischemic risk is identified [66,67]. The exact duration and combination of antithrombotic therapy should be individualized and guided based on the ischemic and bleeding risk profile [60].*

### 3.2. Proton Pump Inhibitors to Decrease Gastrointestinal Bleeding Risk

Proton pump inhibitors (PPI) decrease the risk of upper gastroduodenal bleeding in patients receiving antithrombotic therapy [68,69]. PPI use was evident in the majority of patients in the comparative DAPT trials in older people [48,51]. If a PPI is started to decrease bleeding risk during the DAPT, DAT, or TAT period, attempts should be made to discontinue after this period, if appropriate. Although data is conflicting, some observational studies have suggested possible associations of long-term PPI therapy with vitamin B12 deficiency, pneumonia, fractures, iron deficiency, chronic kidney disease (CKD), and *Clostridioides difficile* infection [70]. If the antiplatelet agent selected is clopidogrel, consideration should also be given to the drug interaction that exists between PPIs and clopidogrel, especially with omeprazole and esomeprazole via CYPC219, and the potential influence on antiplatelet activity, and an alternative PPI should be prescribed [71].

*Recommendation: PPIs should be considered in all older patients receiving DAPT* *, DAT, or TAT who are at high risk of gastrointestinal bleeding. Consideration should be given to avoiding long-term use of PPIs, if possible, with subsequent deprescribing if bleeding risk decreases to an acceptable level.*

### 3.3. Lipid-Lowering Therapy

Intensive lipid-lowering therapy, achieved by the commencement of high-dose statin therapy, is a key goal in secondary prevention after ACS. However, recommendations for older patients differ across guidelines [35,72]. In patients ≥ 75 years of age, a recent meta-analysis has confirmed the efficacy of low-density lipoprotein cholesterol lowering, and a similar risk decrease across all major cardiovascular events was observed between older patients and younger patients [73]. This finding was observed across statin and non-statin (ezetimibe and PCSK9 inhibitor) trials [73]. Where possible, initiation of high-dose, high-intensity statin therapy should be considered in older patients after ACS, and consideration should be paid to likely drug interactions or comorbidities, which may affect prescribing choices (Table 1). If high-intensity statin therapy cannot be tolerated, a moderate-intensity statin combined with ezetimibe appears to have similar cardiovascular benefits in older adults [74]. Myopathy and muscle-associated adverse effects with statin therapy are common and must be considered [75]. The incidence of older patients who encounter statin-induced myopathy and incur harm through increased falls, decreased physical activity, or disability, is not well defined, and further research is required [76]. Concerns regarding statin-associated cognitive decline have been raised in aftermarket reports, but a relationship has not been observed in RCTs [77].

*Recommendation: Commencement of a high-intensity statin in older patients after ACS should be considered unless frailty, comorbidities, and/or drug–drug or drug–disease interactions would favor a decrease in statin dose or intensity (Table 1). After commencement, close monitoring of adverse effects is necessary; if such effects develop, the dose should promptly be decreased to ensure ongoing compliance, given the benefits in this cohort.*

### 3.4. Beta-Blocker Therapy

The evidence supporting the use of beta-blockers after ACS is strongest in patients with decreased left ventricular ejection fraction (LVEF), large anterior MIs, or tachyarrhythmias [35,78]. Routine use of beta-blockers outside this cohort may be considered; however, contemporary observational studies suggest a lack of mortality benefit since the emergence of reperfusion therapy and progressive pharmacotherapy, for which large RCT trials remain ongoing [79,80,81,82,83,84]. Of interest, the recently published results of the REDUCE-AMI trial have demonstrated no significant benefit of beta-blockers among patients who underwent early coronary angiography after MI and had a preserved LVEF [85]. In a recent observational study of 1156 patients ≥ 80 years of age after ACS, beta-blocker use at discharge showed no significant association with a decrease in cardiovascular mortality over a mean follow-up period of 26 months [86]. Beta-blockers may be less tolerated by older adults, with an increased risk of resting conduction disease and noticeable fatigue, in addition to comorbid bronchospasm syndromes [87].

The duration of beta-blocker therapy in older patients after ACS without decreased LVEF, and the timing for deprescribing, remains unknown [35]. A recent observational study in 6893 patients ≥ 65 years of age receiving beta-blockers, compared with patients not receiving beta-blocker therapy, has reported no differences in cardiovascular outcomes beyond 3 years, regardless of dose [88]. Studies in younger patients have suggested the possibility of even earlier cessation and have shown no differences in cardiovascular outcomes at 1 year or earlier, although the existing evidence is conflicting [89,90,91,92,93,94,95,96].

*Recommendation: If beta-blockers are commenced in older patients after ACS, careful consideration must be paid to monitoring for adverse effects such as hypotension, bradycardia, fatigue, or breathlessness; dose reduction or cessation should be considered if quality of life is affected (Table 1) [87].*

### 3.5. Renin–Angiotensin–Aldosterone System Inhibitors

Angiotensin-converting enzyme inhibitors (ACEI) improve outcomes in patients after ACS, particularly patients with an associated decrease in LVEF ≤ 40%, CKD, diabetes, and/or hypertension [35,78]. In older patients with reduced systolic function after ACS, the subset of patients ≥ 65 years of age within the Acute Infarction with Ramipril Efficacy (AIRE) Trial and The Salvage and Ventricular Enlargement (SAVE) Trial showed a significant decrease in mortality with long-term ACEI use [97,98]. Angiotensin receptor blockers (ARB) can be considered an alternative to ACEI therapy in patients unable to tolerate ACEI [99,100,101,102].

In older people, ACEI or ARB may be underused because of concerns regarding complications such as hyperkalemia, worsening renal function, and hypotension [103]. This may be overcome by ensuring close monitoring of blood pressure, serum potassium, and renal function in the first 12 weeks of introduction and at any subsequent dose change [104]. If creatinine clearance increases > 30% from baseline (or eGFR reduction is >25%), the ACEI or ARB dose should be decreased or discontinued [36]. ACEI or ARB initiation after ACS is known to be beneficial but effects extend to prevention and management of nephropathy from diabetes, which is often comorbid in the older population [105].

Evidence suggests that ACEI therapy may improve physical function in older people; however, further research is needed [106,107]. A recent meta-analysis of older patients has indicated that those taking ACEI or ARB that cross the blood-brain barrier (ACEI: captopril, fosinopril, lisinopril, perindopril, ramipril and trandolapril; ARBs: telmisartan and candesartan) have better memory recall at 3 years than observed in patients taking ACEI or ARB that do not cross the blood-brain barrier [108]. Afterload reduction with ACEI and ARB is also thought to be beneficial in the management of aortic valve disease, which often coexists in older patients even if not severe [109].

*Recommendation: ACEI or ARB should be considered in older patients after ACS, especially with left ventricular systolic impairment, and particularly if concurrent comorbidity is present, wherein dual benefits would be obtained; close monitoring of adverse effects is warranted.*

### 3.6. Additional Pharmacotherapy Options

The EPHESUS Trial, investigating the addition of the mineralocorticoid receptor antagonist eplerenone to optimal medical therapy post-ACS, showed a significant decrease in mortality with eplerenone use compared to placebo (RR 0.85; 95% CI 0.75–0.96; *p* = 0.008); however, this finding was not observed in the cohort of patients ≥ 65 years of age [110]. The PARADISE-MI trial, comparing ACEI (ramipril) to the angiotensin receptor-neprilysin inhibitor (sacubitril-valsartan), in patients post-MI, has not shown a significantly lower incidence of death from cardiovascular causes, or incident heart failure with sacubitril-valsartan compared to ramipril (11.9% vs. 13.2%; HR 0.90; 95% CI 0.78–1.04; *p* = 0.17) [111]. Recent observational data suggests improved outcomes with early initiation of sodium–glucose cotransporter 2 inhibitors post-ACS in patients with T2DM; however, further RCTs are required to confirm this benefit and indication in older adults [112,113].

*Recommendation: Continued research to ascertain novel pharmacotherapy to improve morbidity and mortality in older adults post-ACS is required.*

## 4. Invasive Coronary Revascularization

### 4.1. Percutaneous Coronary Intervention

Management for STEMI is consistent across age groups; however, patients ≥ 75 years of age, compared with those < 55 years of age, tend to delay seeking timely medical attention (OR 2.16; 95% CI 1.69–2.77) [114]. Older patients with STEMI, compared to younger patients, typically have more contraindications to invasive revascularization and are less likely to receive reperfusion therapy [115]. Older patients who undergo primary PCI experience a significantly lower overall risk of death, reinfarction, and disabling stroke at 30 days than observed in patients receiving fibrinolysis (14.9% vs. 21.5%; OR 0.64; 95% CI 0.45–0.91; *p* = 0.013) [116]. In a meta-analysis by de Boer et al. comparing PCI and fibrinolysis, patients *>* 80 years of age had a lower incidence of mortality (26.4% vs. 18.3%; *p* = 0.049), stroke (7.9% vs. 5.8%; *p* = 0.45), and repeat MI (7.0% vs. 3.9%; *p* = 0.18) at 30 days than did patients randomized to receive fibrinolysis [117].

For patients presenting with NSTEMI, cardiovascular risk evaluations are used to guide management. The Global Registry of Acute Coronary Events (GRACE) and Thrombolysis in Myocardial Infarction (TIMI) scores are frequently used [118,119]. The 2021 American College of Cardiology/American Heart Association Guideline recommends revascularization for patients with high-risk non-ST-segment elevation ACS (NSTE-ACS) (class 1 recommendation) [36]. Emergent revascularization is advised for patients with cardiogenic shock and unstable patients, including those experiencing refractory angina, hemodynamic, or electrical instability (class 1 recommendation), whereas an early invasive strategy (within 24 hours) is advised for stabilized high-risk patients. Patients classified as intermediate or low-risk are advised to undergo an invasive strategy before discharge (class 2A recommendation). 

Guidelines lack evidence-based recommendations for older people, because of the exclusion of such patients from most major trials. The GRACE score, heavily weighted by age, classifies older adults as having higher risk. Among patients 90–99 years of age, the GRACE score predicts in-hospital mortality, whereas the TIMI score does not [120]. Conventional risk scores do not consider cognitive dysfunction, frailty, comorbidities, and life expectancy [121]. The Geriatric Nutritional Risk Index combined with the Systemic Immunoinflammatory Index outperforms the GRACE score in predicting major adverse cardiovascular events in older people [122].

In a study of 1939 patients ≥ 85 years of age presenting with NSTEMI, fewer than 10% received invasive management [123]. Factors including preserved cognition, independent mobility, and living status were associated with lower in-hospital mortality. Invasive management remained the strongest independent predictor of prolonged survival after adjustment for risk factors (HR 0.47; 95% CI 0.26–0.85; *p* = 0.01). The MOSCA-FRAIL trial of 167 patients ≥ 70 years of age with clinical frailty found no advantage of a routine invasive strategy in terms of days alive and out of the hospital during the first year [124]. A meta-analysis by Reaño et al. including six RCTs with a total of 3768 patients revealed no significant effects of an invasive strategy in decreasing all-cause mortality, cardiovascular mortality, MI, or stroke [125]. In contrast, a meta-analysis by Ma et al. has indicated a decrease in the risk of death with the use of an invasive strategy, during follow-up from 6 months to 5 years (RR 0.65; 95% CI 0.59–0.73, *p* < 0.001), primarily in observational studies [126]. These mixed results highlight the need for individualized assessments in older patients with NSTEMI, considering both clinical frailty and the potential benefits of invasive management. Furthermore, it can be considered reasonable for older patients to have invasive angiography to clarify anatomy but proceed with medical therapy. Likewise, an initial strategy of medical therapy for ACS can be considered a reasonable approach with serial review.

A post hoc analysis of 313 patients ≥ 75 years of age from the Italian Elderly ACS study demonstrated that coronary revascularization was independently associated with a lower risk of mortality in older patients with CKD presenting with NSTE-ACS [127]. Despite this, older age and CKD are strong negative predictors of receiving coronary angiography during the index admission for patients with NSTEMI [6]. CKD is associated with a high prevalence of CAD and is present in up to 43% of patients with ACS [128,129]. Among 1304 patients ≥ 75 years of age who underwent PCI in the Italian Elderly ACS Collaboration studies, 57% had stage 3 CKD, while 11% had stage 4 or 5 CKD at baseline [130]. Patients with CKD experienced significantly higher rates of acute kidney injury (AKI), and patients who developed AKI were at a significantly higher risk of all-cause mortality independent of their baseline renal function. At the 12-month follow-up, patients with stage 4 or 5 CKD had notably higher mortality rates compared to those with stage 3 CKD, evident in both cardiovascular mortality (10.2% vs. 5.2%, *p* < 0.001) and all-cause mortality (17.8% vs. 7.5%, *p* < 0.001). These findings underscore the importance of considering CKD severity in the management of ACS in older patients.

Older adults have a higher prevalence of multivessel disease than younger adults [131]. The FULL REVASC trial has not indicated a lower risk of a composite of death from any cause, MI, or unplanned revascularization in patients undergoing complete revascularization in comparison to a culprit-lesion-only PCI approach [132]. The DANAMI-3-PRIMULTI trial substudy has indicated no difference in outcomes between culprit-only and complete FFR-guided revascularization 27 months after STEMI [133]. The applicability of these trial data to older patients is uncertain, because of the underrepresentation of patients ≥ 75 years of age. Notably, in the FIRE trial, physiology-guided complete revascularization, compared with culprit-lesion-only revascularization, decreased composite outcomes at 1 year (HR 0.73; 95% CI 0.57–0.93; *p* = 0.01) in a cohort of patients ≥ 75 years of age with STEMI or NSTEMI and multivessel disease, thus highlighting the benefits of complete revascularization after ACS, even in an older population [134].

In-hospital adverse outcomes rapidly increase in patients ≥ 80 years of age [135]. In a large Japanese registry study, the risk of adverse outcomes progressively increased with age, such that patients ≥ 90 years of age exhibited the highest in-hospital mortality (OR 3.60; 95% CI 3.10–4.18; *p* < 0.001) and bleeding complications (OR 1.79; 95% CI 1.35–2.36; *p* < 0.001), as compared with patients 60–69 years of age with ACS. Predictors of in-hospital mortality and bleeding complications among patients > 70 years of age included acute heart failure, history of heart failure, female sex, acute MI, cardiogenic shock, CKD, triple-vessel disease, and left main trunk lesions. A transradial PCI approach compared with transfemoral intervention was associated with diminished in-hospital mortality (OR 0.41; 95% CI 0.37–0.45; *p* < 0.001) and bleeding complications (OR 0.38; 95% CI 0.33–0.45; *p* < 0.001), and should be considered the default treatment, particularly in the older population with HBR. 

A subgroup within the older adult population presents with ACS complicated by cardiogenic shock. The prevalence of cardiogenic shock in older adults is notably higher compared to younger patients, both in cases of STEMI (10.8% vs. 3.9%, *p* < 0.0001) and NSTE-ACS (4.6% vs. 1.8%, *p* < 0.0001) [136]. Despite comparable 1-year survival rates between older adults ≥ 70 years of age with acute MI complicated by cardiogenic shock undergoing PCI and patients < 70 years of age, older adults exhibit lower procedural success rates and higher in-hospital mortality [137,138]. Long-term survival among hospital survivors ≥ 70 years of age can be achieved through careful patient selection and the timely implementation of revascularization strategies. The increased long-term mortality observed in older adults compared to younger adults (*p* < 0.01) is primarily attributed to high in-hospital mortality rates (48% vs. 36%, *p* < 0.05). The scientific statement from the American Heart Association recommends that, regardless of age, the initial treatment approach for older patients with cardiogenic shock should prioritize early recognition and stabilization while considering age-related risks and patients’ goals and values [139].

**Figure 4:**
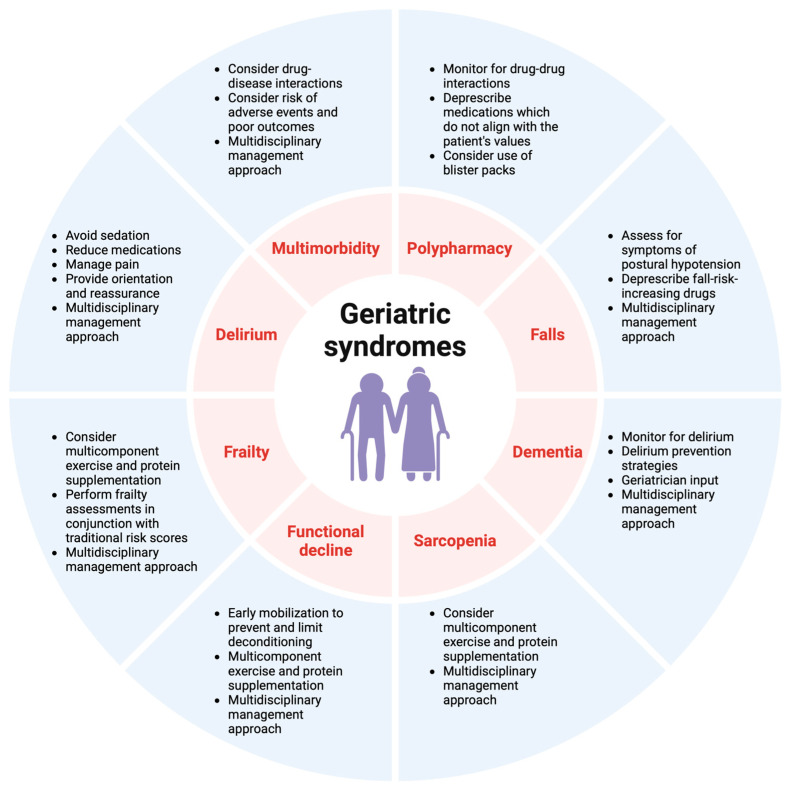
Geriatric syndromes and key management strategies.

Whereas clear evidence is available to support the management of STEMI in older patients, the advantages of routinely implementing an invasive approach in older patients with NSTEMI are less evident. Conflicting literature results have contributed to uncertainty regarding optimal management strategies. When evaluating revascularization strategies for older patients, clinicians should consider additional patient preferences, bleeding risk, geriatric syndromes (Figure 4), and comorbidities, which may influence life expectancy and treatment decisions. A shared decision-making process should be considered with the patient and their family.

### 4.2. Surgical Revascularization

The 2021 American College of Cardiology/American Heart Association Guideline for Coronary Artery Revascularization recommends CABG for patients with significant left main CAD and high-complexity CAD, to improve survival over that achieved with PCI (class 1 recommendation) [36]. CABG is also recommended for patients with multivessel CAD and significant anatomical complexity, as categorized by a Syntax score > 33, because of survival advantages (class 2A recommendation). Additionally, CABG is recommended for patients with diabetes and multivessel CAD involving the left anterior descending artery, to decrease mortality and the need for repeat revascularization. Most RCTs comparing CABG and PCI in older patients have focused on stable CAD, whereas limited evidence is available regarding the role of CABG in older adults with ACS. 

In patients ≥ 65 years of age with ACS and multivessel CAD, CABG is associated with a significantly greater number of days alive and out of the hospital, and a higher probability of achieving ≥ 90% of days alive and out of the hospital at both 3 and 5 years [140]. CABG, compared with PCI, significantly prolongs survival (*p* < 0.0001) and is associated with decreases in the incidence of all-cause mortality (HR 0.80; 95% CI 0.73–0.88), repeat coronary revascularization (HR 0.63; 95% CI 0.58–0.69), rehospitalization (HR 0.89; 95% CI, 0.84–0.95, and nonfatal MI (HR 0.83; 95% CI, 0.77–0.90), but not nonfatal stroke (0.93; 95% CI, 0.85–1.01).

A retrospective cohort study in 597 patients ≥ 75 years of age with multivessel or left main CAD reported higher mortality in patients who underwent PCI compared to those who underwent CABG (39.9% vs. 25.4%, *p* < 0.001) at the 5-year follow-up [141]. In this cohort, 75% of patients who underwent PCI had stable CAD (vs. 25% with ACS), while 83% of patients who underwent CABG had stable CAD (vs. 17% with ACS). Patients who underwent emergency revascularization or presented with STEMI were excluded. After adjustment for older age, higher creatinine, and left main disease, recurrent ACS (aHR 2.20; 95% CI 1.23–3.96; *p* = 0.008) and repeat revascularization (aHR 2.54; 95% CI 1.36–4.73; *p* = 0.003) occurred significantly more frequently in patients who underwent PCI rather than CABG; in contrast, bleeding (aHR 0.10; 95% CI 0.02–0.53; *p* = 0.007) and new-onset AF (aHR 0.40; 95% CI 0.20–0.79, *p* = 0.008) were significantly more common in patients who underwent CABG. Among older patients hospitalized with CAD, those 80–89 years of age, compared with those 70–79 years of age, exhibit a higher likelihood of developing cardiac complications (OR 1.20; 95% CI 1.12–1.23), renal complications (OR 1.54; 95% CI 1.48–1.61; *p* < 0.001), infectious complications (OR 1.41; 95% CI 1.34–1.48; *p* < 0.001), and respiratory complications (OR 1.2; 95% CI 1.2–2.1; *p* < 0.001), thus leading to poorer surgical outcomes and higher mortality rates (OR 1.41; 95% CI 1.36–1.61, *p* < 0.001) [142]. Ultimately, decision-making for revascularization in older patients with ACS is challenging, because these patients often have high anatomical complexity but are perceived to benefit less from CABG than younger patients, because of the higher periprocedural morbidity and mortality associated with the surgery.

## 5. Non-Pharmacological Interventions, Cardiac Rehabilitation, and the Multidisciplinary Team

Managing older patients with frailty and comorbidities requires careful use of non-pharmacological interventions and comprehensive discharge planning involving patients, their families, and MDT discussions. Frailty should be routinely assessed as it significantly predicts adverse outcomes in older patients with CAD [143]. Non-pharmacological interventions are crucial in the care of older patients after ACS, who have high long-term mortality rates (65% 8-year mortality rate for those ≥ 65 years of age) [3]. The 30-day readmission rate for patients ≥ 70 years of age is almost 25%, and the 1-year readmission rate is approximately 60% [144].

Cardiac rehabilitation (CR) integrates exercise, education, counseling, and modification of cardiac risk factors [145]. CR offers personalized approaches considering frailty, medication adherence, cognitive dysfunction, and comorbidities. This therapy decreases mortality rates by 21–34% in older patients after ACS or revascularization procedures [146]. Despite its benefits, only 25% of eligible Medicare beneficiaries eligible for CR participate, of whom only 27% complete the program [147]. Patients attending 36 sessions have a 47% lower risk of mortality and a 31% lower risk of MI than those completing only one session [148].

Delirium, which frequently occurs during the hospitalization of older people, increases mortality and poor outcomes [149]. Its incidence in patients ≥ 75 years of age admitted for acute cardiac diseases is 17.2%, thus leading to prolonged hospitalization, functional decline, and a four-fold higher 12-month mortality risk [150]. Delirium is preventable in 30–40% of cases through reorientation strategies, environmental modifications, the use of visual and hearing aids, and the avoidance of precipitating medications such as benzodiazepines [149]. In a study of 908 patients ≥ 70 years of age by Tonet et al., patients who were malnourished or at high risk of malnutrition had a 50% mortality rate over a 288-day follow-up period [151]. Medication non-adherence among older patients is also associated with an elevated risk of hospitalization and mortality, and a decreased quality of life [152].

The MDT, including cardiologists, geriatricians, general and internal medicine physicians, nurses, physiotherapists, occupational therapists, psychologists, pharmacists, dieticians, social workers, and primary care physicians can optimize holistic, patient-centered care after ACS [34]. The MDT can improve outcomes in this population by maintaining function, decreasing deconditioning, rationalizing medications, achieving aggressive risk factor reduction, and decreasing delirium risk [33]. Comprehensive MDT management at discharge can help decrease readmission, morbidity, and mortality in older patients. The MDT can ensure that home setups are optimal for maintaining function and mitigating the risk of falls and provides additional education to patients and their families regarding diagnosis, medications, nutrition, and physical activity, as well as detailed instructions regarding follow-up and CR. Given the comorbidities, frailty, cognitive decline, and disabilities in older patients after ACS, the MDT can also ensure that interventions align with patient values and goals of care. Consideration of a patient’s values should be given when deciding between invasive and non-invasive strategies, particularly when an older patient’s values focus on quality of life as opposed to life-prolonging measures. The primary focus in the management of older patients with ACS involves transitioning from a disease-centered model to a person-centered approach.

## 6. Myocardial Infarction in the Context of Non-Cardiac Surgeries in Older People

The prevalence of patients > 75 years of age undergoing intermediate or high-risk non-cardiac surgery (NCS) is increasing [153]. Age is a known risk factor for adverse cardiovascular events after NCS, including perioperative MI, and patients ≥ 75 years of age have a significantly greater risk than younger adults (9.5% vs. 4.8%; *p* < 0.001) [154].

In a study of older patients > 70 years of age who underwent hip fracture surgery, in-hospital and 1-year mortality rates were significantly higher in patients with perioperative myocardial injury and/or infarction (PMI: hs-cTn T/I above the 99th percentile of the upper reference value) than in patients without evidence of myocardial injury [155,156]. PMI may encompass both ischemic and non-ischemic causes of myocardial injury, such as PE or sepsis. Myocardial injury after non-cardiac surgery (MINS) within 30 days of NCS is limited to ischemic causes of PMI [157]. In cases of PMI, analgesia or anesthesia may mask symptoms of ischemia, and a lack of postoperative ECG and biomarker monitoring may increase the risk of missed diagnosis, particularly as 90% of PMI events are minimally symptomatic [157]. Alternatively, PMI may be misattributed to postoperative pain, nausea, surgical wounds, or drains. Therefore, preoperative risk stratification and postoperative surveillance in older patients after intermediate or high-risk NCS are essential [158,159].

Preoperatively, in patients undergoing intermediate or high-risk NCS aged ≥ 65 years of age, in addition to a thorough history, a physical examination, standard laboratory tests, an ECG and troponin (class 1 recommendation), a B-type natriuretic peptide or N-terminal pro-B-type natriuretic peptide (class 2A recommendation), and a functional capacity assessment should be performed [153]. It is important to recognize that diseases other than heart failure may cause elevation in natriuretic peptide levels, and higher cut-offs may need to be considered as a result [160]. Consideration should be given to transthoracic echocardiography, and/or stress imaging for CAD, in patients with positive biomarkers in the absence of known disease [161]. Risk stratification tools such as the Revised Cardiac Risk Index, Surgical Risk Calculator, The American College of Surgery National Surgical Quality Improvement Program, the Surgical Outcome Risk Tool, or The American University of Beirut-HAS2 Cardiovascular Risk Index can further identify patients at high risk of postoperative cardiovascular complications [153]. In older patients, assessing frailty and function is crucial, because poorer function and frailty are predictive of adverse postoperative outcomes [162,163]. If the preoperative assessment suggests that surgery would pose a high risk for an older patient, consultation in an MDT forum, including cardiologists and geriatricians, should be considered, with an emphasis on aligning with the patient’s goals of care, values, and preferences, and ensuring that shared decision-making is achieved regarding the risks and possible adverse outcomes, before surgical intervention proceeds. Although data in the older population are lacking, the continuation of aspirin in patients with prior PCI undergoing NCS should be considered unless there is a very high perioperative bleeding risk [164]. 

Postoperatively, serial ECG and troponin monitoring on at least postoperative days 1 and 2 should be considered to identify PMI in high-risk older patients and ascertain the need for further assessment or intervention [162,163]. Consideration should also be given to the appropriateness of the care environment, with postoperative referral to high-dependency or intensive care units depending on the individual risk profile.

## 7. Conclusions

Managing ACS in older adults presents unique challenges, because of age-related physiological changes, the prevalence of geriatric syndromes, patient goals of care, and frailty, in addition to understanding the risk–benefit ratio of management strategies. Addressing these issues requires further research, as well as the adaptation of guidelines to bridge existing gaps and meet the evolving challenges of this growing demographic. This review describes a comprehensive and integrated approach to managing ACS in older patients, covering the complexities of the diagnostic workup, treatment modalities, pharmacotherapy, and after ACS care, as well as considering PMI in older patients after noncardiac surgery. By addressing the notable gaps in existing guidelines, this guide is aimed at providing clinicians with a concise, yet comprehensive overview of the complexities involved in ACS management in older populations.

## Figures and Tables

**Table 1:** Summary of pharmacotherapy post-acute coronary syndrome and considerations in the older population.

Drug Class ^†^ and Mechanism of Action	Side Effects	Drug–Disease Interactions (Side Effects)	Drug–Drug Interactions (Side Effects)	Other Considerations in the Older Population	Duration of Use
**Antiplatelet therapy** **Aspirin** Irreversibly binds to cyclo-oxygenase, reducing the synthesis of thromboxane A_2_ (inducer of platelet aggregation)	GI ulceration, bleeding, skin reactions	Gastritis or peptic ulcer disease, bleeding disorders, severe hepatic disease (↑ bleeding risk), asthma (bronchospasm)	Corticosteroids, NSAIDs (↑risk of gastric ulceration) Anticoagulants (↑ bleeding)	Continued assessment of bleeding risk through risk stratification, reviewing concurrent comorbidities and medications	Long-term *
**P2Y_12_ inhibitor therapy** **Prasugrel, Clopidogrel, Ticagrelor** Binds irreversibly (prasugrel, clopidogrel) or reversibly (ticagrelor) to the P2Y12 receptor inhibiting platelet aggregation	GI ulceration, bleeding *Ticagrelor*—dyspnea, non-cardiac chest pain, raised uric acid concentration	Bleeding disorders, severe hepatic disease (↑ bleeding risk) *Prasugrel—*previous stroke or TIA (↑ bleeding risk and risk of hemorrhagic strokes)	*Clopidogrel* CYP2C19 heterogenicity—poor metabolizers (↓ efficacy), PPIs—omeprazole, esomeprazole (↓ efficacy) *Ticagrelor*—CYP3A4 inhibitors (↑ bleeding)	Continued assessment of bleeding risk through risk stratification, reviewing concurrent comorbidities and medications	Figure 3
**Lipid-lowering therapy** **Atorvastatin, Rosuvastatin (high-intensity)** HMG-CoA reductase inhibitors (statins) increases hepatic cholesterol uptake from the blood. Atherosclerotic lesion/plaque stabilization	Myalgia and myopathy, sleep disturbance (insomnia, nightmares), liver toxicity	Chronic liver disease diabetes, CKD, (statin -induced myopathy)	Atorvastatin—dose reduction with CYP3A4 inhibitors	Close monitoring of side effects as myopathies may impact on physical function, frailty and falls and sleep disturbances may impact on risk of delirium and falls	Long-term *
**Beta-blocker therapy** Reduces heart rate and blood pressure, modulates myocardial contractility and oxygen demand, can improve myocardial ischemia and reduce ventricular arrythmias	Bradycardia, hypotension, fatigue, bronchospasm, claudication, masking symptoms of hypoglycemia and depression	COPD or asthma (bronchospasm), diabetes (hypoglycemia), renal impairment, AV node dysfunction (bradycardia), PVD (claudication)	Gliclazides (hypoglycemia), AV nodal blocking medications (bradycardia)	Close monitoring of side effects as hypotension and bradycardia may increase falls risk, fatigue may decrease function and independence	Consider cessation at 3 years or sooner
**ACEI and ARBs** Inhibition of the angiotensin converting enzyme or angiotensin receptor blocker	Hypotension, hyperkalemia, worsening renal function, angioedema, cough (ACEI)	CKD (hyperkalemia and renal failure)	Trimethoprim, ciclosporin (increase potassium concentration) NSAIDs, diuretics (impaired renal function)	Close monitoring of side effects as hypotension can increase falls risk	Long-term *

* Long-term: Defined as a time course in which medications that are life-prolonging are continued in alignment with the patient’s current values, preferences, and overall prognosis. If these values and preferences change or overall prognosis becomes poor, these medications should be reviewed and deprescribed as appropriate. In addition, if any harm or adverse effects occur then they should be discontinued. † Bold text identifies the drug classes and examples of drugs within the drug classes where relevant. ↓: refers to decreased, ↑: refers to increased, ACEI: angiotensin-converting enzyme inhibitor; ARBs: angiotensin receptor blocker; AV: atrioventricular; CCB: calcium channel blockers; CKD: chronic kidney disease; COPD: chronic obstructive pulmonary disease; eg: for example; GI: gastrointestinal; HFrEF: heart failure with reduced ejection fraction defined as left ventricular ejection fraction ≤ 40%; HMG-CoA: 3-Hydroxy-3-methylglutaryl coenzyme A; NSAIDs: non-steroidal anti-inflammatories; PPI: proton pump inhibitor, PVD: peripheral vascular disease; TIA: transient ischemic attack.

**Table 2:** ARC-HBR Criteria for assessing high bleeding risk at time of PCI.

Major Criteria	Minor Criteria
Anticipated use of long-term oral anticoagulation Severe or end-stage CKD (eGFR < 30 mL/min) Hemoglobin < 11 g/dL Spontaneous bleeding requiring hospitalization or transfusion in the past 6 months or at any time, if recurrent Moderate or severe baseline thrombocytopenia (platelet count < 100 × 10^9^/L) Chronic bleeding diathesis Liver cirrhosis with portal hypertension Active malignancy within the last 12 months (excluding non-melanoma skin cancer) Previous spontaneous ICH (at any time), previous traumatic ICH within the past 12 months, presence of brain AVM, moderate or severe ischemic stroke within the past 6 months Non-deferrable major surgery on DAPT Recent major surgery or major trauma within 30 days before PCI	Age > 75 years of age Moderate CKD (eGFR 30–59 mL/min) Hemoglobin 11–12.9 g/dL for men and 11–11.9 g/dL for women Spontaneous bleeding requiring hospitalization or transfusion within the past 12 months not meeting the major criterion Long-term use of oral NSAIDs or steroids Any ischemic stroke at any time not meeting the major criterion

ARC-HBR: Academic Research Consortium for High Bleeding Risk; AVM: arteriovenous malformation; CKD: chronic kidney disease; DAPT: dual antiplatelet therapy; eGFR: estimated glomerular filtration rate; ICH: intracranial hemorrhage; NSAID: non-steroidal anti-inflammatory drug; PCI: percutaneous coronary intervention.

## Data Availability

Not applicable.

## References

[1] United Nations Department of Economic and Social Affairs (2022). United Nations Department of Economic and Social. Affairs, Population Division. 2022. World Population Prospects 2022: Summary of Results.

[2] Gulati M., Levy P.D., Mukherjee D., Amsterdam E., Bhatt D.L., Birtcher K.K., Blankstein R., Boyd J., Bullock-Palmer R.P., Conejo T. (2021). 2021 AHA/ACC/ASE/CHEST/SAEM/SCCT/SCMR Guideline for the Evaluation and Diagnosis of Chest Pain: A Report of the American College of Cardiology/American Heart Association Joint Committee on Clinical Practice Guidelines. Circulation.

[3] Kochar A., Chen A.Y., Sharma P.P., Pagidipati N.J., Fonarow G.C., Cowper P.A., Roe M.T., Peterson E.D., Wang T.Y. (2018). Long-Term Mortality of Older Patients With Acute Myocardial Infarction Treated in US Clinical Practice. J. Am. Heart Assoc..

[4] Ariza-Solé A., Alegre O., Elola F.J., Fernández C., Formiga F., Martínez-Sellés M., Bernal J.L., Segura J.V., Iñíguez A., Bertomeu V. (2019). Management of myocardial infarction in the elderly. Insights from Spanish Minimum Basic Data Set. Eur. Heart J. Acute Cardiovasc. Care.

[5] Alexander K.P., Roe M.T., Chen A.Y., Lytle B.L., Pollack C.V., Foody J.M., Boden W.E., Smith S.C., Gibler W.B., Ohman E.M. (2005). Evolution in cardiovascular care for elderly patients with non-ST-segment elevation acute coronary syndromes: Results from the CRUSADE National Quality Improvement Initiative. J. Am. Coll. Cardiol..

[6] De Luca L., Olivari Z., Bolognese L., Lucci D., Gonzini L., Di Chiara A., Casella G., Chiarella F., Boccanelli A., Di Pasquale G. (2014). A decade of changes in clinical characteristics and management of elderly patients with non-ST elevation myocardial infarction admitted in Italian cardiac care units. Open Heart.

[7] Ferrucci L., Fabbri E. (2018). Inflammageing: Chronic inflammation in ageing, cardiovascular disease, and frailty. Nat. Rev. Cardiol..

[8] Lee P.Y., Alexander K.P., Hammill B.G., Pasquali S.K., Peterson E.D. (2001). Representation of Elderly Persons and Women in Published Randomized Trials of Acute Coronary Syndromes. JAMA.

[9] Sanchez-Nadales A., Igbinomwanhia E., Grimm R.A., Griffin B.P., Kapadia S.R., Xu B. (2023). Contemporary Trends in Clinical Characteristics, Therapeutic Strategies and Outcomes in Patients Aged 80 Years and Older Presenting with non-ST Elevation Myocardial Infarctions in the United States. Curr. Probl. Cardiol..

[10] Thygesen K., Alpert J.S., Jaffe A.S., Chaitman B.R., Bax J.J., Morrow D.A., White H.D. (2018). Fourth Universal Definition of Myocardial Infarction (2018). J. Am. Coll. Cardiol..

[11] Hwang S.Y., Park E.H., Shin E.S., Jeong M.H. (2009). Comparison of factors associated with atypical symptoms in younger and older patients with acute coronary syndromes. J. Korean Med. Sci..

[12] Nanna M.G., Hajduk A.M., Krumholz H.M., Murphy T.E., Dreyer R.P., Alexander K.P., Geda M., Tsang S., Welty F.K., Safdar B. (2019). Sex-Based Differences in Presentation, Treatment, and Complications Among Older Adults Hospitalized for Acute Myocardial Infarction: The SILVER-AMI Study. Circ. Cardiovasc. Qual. Outcomes.

[13] Bruckenthal P., Fillit H.M., Rockwood K., Young J. (2017). Pain in the Older Adult. Brocklehurst's Textbook of Geriatric Medicine and Gerontology.

[14] Hung C.-L., Hou C.J.-Y., Yeh H.-I., Chang W.-H. (2010). Atypical Chest Pain in the Elderly: Prevalence, Possible Mechanisms and Prognosis. Int. J. Gerontol..

[15] Kaze A.D., Fonarow G.C., Echouffo-Tcheugui J.B. (2023). Cardiac Autonomic Dysfunction and Risk of Silent Myocardial Infarction Among Adults with Type 2 Diabetes. J. Am. Heart Assoc..

[16] Koshy A.N., Dinh D.T., Fulcher J., Brennan A.L., Murphy A.C., Duffy S.J., Reid C.M., Ajani A.E., Freeman M., Hiew C. (2022). Long-term mortality in asymptomatic patients with stable ischemic heart disease undergoing percutaneous coronary intervention. Am. Heart J..

[17] Friedman A., Chudow J., Merritt Z., Shulman E., Fisher J.D., Ferrick K.J., Krumerman A. (2020). Electrocardiogram abnormalities in older individuals by race and ethnicity. J. Electrocardiol..

[18] Pope J.H., Ruthazer R., Kontos M.C., Beshansky J.R., Griffith J.L., Selker H.P. (2004). The impact of electrocardiographic left ventricular hypertrophy and bundle branch block on the triage and outcome of ED patients with a suspected acute coronary syndrome: A multicenter study. Am. J. Emerg. Med..

[19] Covino M., Simeoni B., Montalto M., Burzotta F., Buccelletti F., Carbone L., Gallo A., Gentiloni Silveri N. (2012). Reduced performance of Troponin T for acute coronary syndromes diagnosis in the elderly and very elderly patients: A retrospective study of 2688 patients. Eur. Rev. Med. Pharmacol. Sci..

[20] Wang A.Z., Schaffer J.T., Holt D.B., Morgan K.L., Hunter B.R. (2020). Troponin Testing and Coronary Syndrome in Geriatric Patients With Nonspecific Complaints: Are We Overtesting?. Acad. Emerg. Med..

[21] Sedighi S.M., Fulop T., Mohammadpour A., Nguyen M., Prud’Homme P., Khalil A. (2021). Elevated Cardiac Troponin Levels in Geriatric Patients Without ACS: Role of Comorbidities. CJC Open.

[22] Kloska S.M., Kozinski M., Stefanska A., Bergmann K., Mankowska-Cyl A., Siodmiak J., Sypniewska G., Krintus M. (2023). Reference values and biological determinants for cardiac myosin-binding protein C concentrations assessed with an enzyme-linked immunosorbent assay. J. Med. Biochem..

[23] Putot A., Derrida S.B., Zeller M., Avondo A., Ray P., Manckoundia P., Cottin Y. (2018). Short-Term Prognosis of Myocardial Injury, Type 1, and Type 2 Myocardial Infarction in the Emergency Unit. Am. J. Med..

[24] Sarkisian L., Saaby L., Poulsen T.S., Gerke O., Jangaard N., Hosbond S., Diederichsen A.C., Thygesen K., Mickley H. (2016). Clinical Characteristics and Outcomes of Patients with Myocardial Infarction, Myocardial Injury, and Nonelevated Troponins. Am. J. Med..

[25] Putot A., Jeanmichel M., Chague F., Manckoundia P., Cottin Y., Zeller M. (2020). Type 2 Myocardial Infarction: A Geriatric Population-based Model of Pathogenesis. Aging Dis..

[26] Forman D.E., de Lemos J.A., Shaw L.J., Reuben D.B., Lyubarova R., Peterson E.D., Spertus J.A., Zieman S., Salive M.E., Rich M.W. (2020). Cardiovascular Biomarkers and Imaging in Older Adults: JACC Council Perspectives. J. Am. Coll. Cardiol..

[27] Ennezat P.V., Logeart D., Berrebi A., Vincentelli A., Maréchaux S. (2010). Key role of Doppler echocardiography in the emergency management of elderly patients. Arch. Cardiovasc. Dis..

[28] Gueret P., Khalife K., Jobic Y., Fillipi E., Isaaz K., Tassan-Mangina S., Baixas C., Motreff P., Meune C. (2008). Echocardiographic assessment of the incidence of mechanical complications during the early phase of myocardial infarction in the reperfusion era: A French multicentre prospective registry. Arch. Cardiovasc. Dis..

[29] Lyon A.R., Bossone E., Schneider B., Sechtem U., Citro R., Underwood S.R., Sheppard M.N., Figtree G.A., Parodi G., Akashi Y.J. (2016). Current state of knowledge on Takotsubo syndrome: A Position Statement from the Taskforce on Takotsubo Syndrome of the Heart Failure Association of the European Society of Cardiology. Eur. J. Heart Fail..

[30] Citro R., Rigo F., Ciampi Q., D'Andrea A., Provenza G., Mirra M., Giudice R., Silvestri F., Di Benedetto G., Bossone E. (2011). Echocardiographic assessment of regional left ventricular wall motion abnormalities in patients with tako-tsubo cardiomyopathy: Comparison with anterior myocardial infarction. Eur. J. Echocardiogr..

[31] Onnis C., Muscogiuri G., Cademartiri F., Fanni D., Faa G., Gerosa C., Mannelli L., Suri J.S., Sironi S., Montisci R. (2023). Non-invasive coronary imaging in elderly population. Eur. J. Radiol..

[32] Madhavan M.V., Gersh B.J., Alexander K.P., Granger C.B., Stone G.W. (2018). Coronary Artery Disease in Patients ≥80 Years of Age. J. Am. Coll. Cardiol..

[33] Damluji A.A., Forman D.E., Wang T.Y., Chikwe J., Kunadian V., Rich M.W., Young B.A., Page R.L., DeVon H.A., Alexander K.P. (2023). Management of Acute Coronary Syndrome in the Older Adult Population: A Scientific Statement From the American Heart Association. Circulation.

[34] Morici N., De Servi S., De Luca L., Crimi G., Montalto C., De Rosa R., De Luca G., Rubboli A., Valgimigli M., Savonitto S. (2022). Management of acute coronary syndromes in older adults. Eur. Heart J..

[35] Byrne R.A., Rossello X., Coughlan J.J., Barbato E., Berry C., Chieffo A., Claeys M.J., Dan G.-A., Dweck M.R., Galbraith M. (2023). 2023 ESC Guidelines for the management of acute coronary syndromes: Developed by the task force on the management of acute coronary syndromes of the European Society of Cardiology (ESC). Eur. Heart J..

[36] Lawton J.S., Tamis-Holland J.E., Bangalore S., Bates E.R., Beckie T.M., Bischoff J.M., Bittl J.A., Cohen M.G., DiMaio J.M., Don C.W. (2022). 2021 ACC/AHA/SCAI Guideline for Coronary Artery Revascularization: Executive Summary: A Report of the American College of Cardiology/American Heart Association Joint Committee on Clinical Practice Guidelines. Circulation.

[37] Salazar J.A., Poon I., Nair M. (2007). Clinical consequences of polypharmacy in elderly: Expect the unexpected, think the unthinkable. Expert. Opin. Drug Saf..

[38] Smaje A., Weston-Clark M., Raj R., Orlu M., Davis D., Rawle M. (2018). Factors associated with medication adherence in older patients: A systematic review. Aging Med..

[39] Maher R.L., Hanlon J., Hajjar E.R. (2014). Clinical consequences of polypharmacy in elderly. Expert. Opin. Drug Saf..

[40] De Servi S., Landi A., Savonitto S., Morici N., De Luca L., Montalto C., Crimi G., De Rosa R., De Luca G. (2023). Antiplatelet Strategies for Older Patients with Acute Coronary Syndromes: Finding Directions in a Low-Evidence Field. J. Clin. Med..

[41] Costa F., van Klaveren D., James S., Heg D., Räber L., Feres F., Pilgrim T., Hong M.K., Kim H.S., Colombo A. (2017). Derivation and validation of the predicting bleeding complications in patients undergoing stent implantation and subsequent dual antiplatelet therapy (PRECISE-DAPT) score: A pooled analysis of individual-patient datasets from clinical trials. Lancet.

[42] Montalto C., Crimi G., Morici N., Palmerini T., Valgimigli M., Savonitto S., De Servi S. (2021). Validation and Additive Predictive Value of the Academic Research Consortium-High Bleeding Risk Criteria in Older Adults. Thromb. Haemost..

[43] Angiolillo D.J., Bhatt D.L., Cannon C.P., Eikelboom J.W., Gibson C.M., Goodman S.G., Granger C.B., Holmes D.R., Lopes R.D., Mehran R. (2021). Antithrombotic Therapy in Patients With Atrial Fibrillation Treated With Oral Anticoagulation Undergoing Percutaneous Coronary Intervention: A North American Perspective: 2021 Update. Circulation.

[44] Crimi G., Morici N., Ferrario M., Ferri L.A., Piatti L., Grosseto D., Cacucci M., Mirizzi A.M., Toso A., Piscione F. (2019). Time Course of Ischemic and Bleeding Burden in Elderly Patients With Acute Coronary Syndromes Randomized to Low-Dose Prasugrel or Clopidogrel. J. Am. Heart Assoc..

[45] Wallentin L., Becker R.C., Budaj A., Cannon C.P., Emanuelsson H., Held C., Horrow J., Husted S., James S., Katus H. (2009). Ticagrelor versus Clopidogrel in Patients with Acute Coronary Syndromes. N. Engl. J. Med..

[46] Wiviott S.D., Braunwald E., McCabe C.H., Montalescot G., Ruzyllo W., Gottlieb S., Neumann F.-J., Ardissino D., De Servi S., Murphy S.A. (2007). Prasugrel versus Clopidogrel in Patients with Acute Coronary Syndromes. N. Engl. J. Med..

[47] Husted S., James S., Becker R.C., Horrow J., Katus H., Storey R.F., Cannon C.P., Heras M., Lopes R.D., Morais J. (2012). Ticagrelor Versus Clopidogrel in Elderly Patients With Acute Coronary Syndromes. Circ. Cardiovasc. Qual. Outcomes.

[48] Gimbel M., Qaderdan K., Willemsen L., Hermanides R., Bergmeijer T., de Vrey E., Heestermans T., Tjon Joe Gin M., Waalewijn R., Hofma S. (2020). Clopidogrel versus ticagrelor or prasugrel in patients aged 70 years or older with non-ST-elevation acute coronary syndrome (POPular AGE): The randomised, open-label, non-inferiority trial. Lancet.

[49] Szummer K., Montez-Rath M.E., Alfredsson J., Erlinge D., Lindahl B., Hofmann R., Ravn-Fischer A., Svensson P., Jernberg T. (2020). Comparison Between Ticagrelor and Clopidogrel in Elderly Patients With an Acute Coronary Syndrome. Circulation.

[50] Bangalore S. (2018). Prasugrel in the Elderly. Circulation.

[51] Savonitto S., Ferri L.A., Piatti L., Grosseto D., Piovaccari G., Morici N., Bossi I., Sganzerla P., Tortorella G., Cacucci M. (2018). Comparison of Reduced-Dose Prasugrel and Standard-Dose Clopidogrel in Elderly Patients With Acute Coronary Syndromes Undergoing Early Percutaneous Revascularization. Circulation.

[52] Roe M.T., Goodman S.G., Ohman E.M., Stevens S.R., Hochman J.S., Gottlieb S., Martinez F., Dalby A.J., Boden W.E., White H.D. (2013). Elderly Patients With Acute Coronary Syndromes Managed Without Revascularization. Circulation.

[53] Koshy A.N., Giustino G., Sartori S., Hooda A., Feng Y., Snyder C., Dasgupta S., Kumar K.R., Krishnamoorthy-Melarcode P., Sweeny J. (2023). Ticagrelor or prasugrel versus clopidogrel in patients undergoing percutaneous coronary intervention for chronic coronary syndromes. EuroIntervention.

[54] Capodanno D., Angiolillo D.J. (2023). Timing, Selection, Modulation, and Duration of P2Y(12) Inhibitors for Patients With Acute Coronary Syndromes Undergoing PCI. JACC Cardiovasc. Interv..

[55] Kim B.K., Hong S.J., Cho Y.H., Yun K.H., Kim Y.H., Suh Y., Cho J.Y., Her A.Y., Cho S., Jeon D.W. (2020). Effect of Ticagrelor Monotherapy vs Ticagrelor With Aspirin on Major Bleeding and Cardiovascular Events in Patients With Acute Coronary Syndrome: The TICO Randomized Clinical Trial. JAMA.

[56] Watanabe H., Morimoto T., Natsuaki M., Yamamoto K., Obayashi Y., Ogita M., Suwa S., Isawa T., Domei T., Yamaji K. (2022). Comparison of Clopidogrel Monotherapy After 1 to 2 Months of Dual Antiplatelet Therapy With 12 Months of Dual Antiplatelet Therapy in Patients With Acute Coronary Syndrome: The STOPDAPT-2 ACS Randomized Clinical Trial. JAMA Cardiol..

[57] Hahn J.Y., Song Y.B., Oh J.H., Cho D.K., Lee J.B., Doh J.H., Kim S.H., Jeong J.O., Bae J.H., Kim B.O. (2018). 6-month versus 12-month or longer dual antiplatelet therapy after percutaneous coronary intervention in patients with acute coronary syndrome (SMART-DATE): A randomised, open-label, non-inferiority trial. Lancet.

[58] Fujisaki T., Kuno T., Iwagami M., Miyamoto Y., Takagi H., Deharo P., Cuisset T., Briasoulis A., Panaich S., Latib A. (2023). Net clinical benefit of dual antiplatelet therapy in elderly patients with acute coronary syndrome: A systematic review and meta-analysis. Catheter. Cardiovasc. Interv..

[59] D’Ascenzo F., De Filippo O., Gallone G., Mittone G., Deriu M.A., Iannaccone M., Ariza-Solé A., Liebetrau C., Manzano-Fernández S., Quadri G. (2021). Machine learning-based prediction of adverse events following an acute coronary syndrome (PRAISE): A modelling study of pooled datasets. Lancet.

[60] Volgman A.S., Nair G., Lyubarova R., Merchant F.M., Mason P., Curtis A.B., Wenger N.K., Aggarwal N.T., Kirkpatrick J.N., Benjamin E.J. (2022). Management of Atrial Fibrillation in Patients 75 Years and Older: JACC State-of-the-Art Review. J. Am. Coll. Cardiol..

[61] Dewilde W.J., Oirbans T., Verheugt F.W., Kelder J.C., De Smet B.J., Herrman J.P., Adriaenssens T., Vrolix M., Heestermans A.A., Vis M.M. (2013). Use of clopidogrel with or without aspirin in patients taking oral anticoagulant therapy and undergoing percutaneous coronary intervention: An open-label, randomised, controlled trial. Lancet.

[62] Gibson C.M., Mehran R., Bode C., Halperin J., Verheugt F.W., Wildgoose P., Birmingham M., Ianus J., Burton P., van Eickels M. (2016). Prevention of Bleeding in Patients with Atrial Fibrillation Undergoing PCI. N. Engl. J. Med..

[63] Cannon C.P., Lip G.Y.H., Oldgren J. (2018). Dual Antithrombotic Therapy with Dabigatran after PCI in Atrial Fibrillation. N. Engl. J. Med..

[64] Lopes R.D., Heizer G., Aronson R., Vora A.N., Massaro T., Mehran R., Goodman S.G., Windecker S., Darius H., Li J. (2019). Antithrombotic Therapy after Acute Coronary Syndrome or PCI in Atrial Fibrillation. N. Engl. J. Med..

[65] Vranckx P., Valgimigli M., Eckardt L., Tijssen J., Lewalter T., Gargiulo G., Batushkin V., Campo G., Lysak Z., Vakaliuk I. (2019). Edoxaban-based versus vitamin K antagonist-based antithrombotic regimen after successful coronary stenting in patients with atrial fibrillation (ENTRUST-AF PCI): A randomised, open-label, phase 3b trial. Lancet.

[66] Menditto A., Antonicelli R. (2020). Is dual therapy the correct strategy in frail elderly patients with atrial fibrillation and acute coronary syndrome?. J. Geriatr. Cardiol..

[67] Yasuda S., Kaikita K., Akao M., Ako J., Matoba T., Nakamura M., Miyauchi K., Hagiwara N., Kimura K., Hirayama A. (2019). Antithrombotic Therapy for Atrial Fibrillation with Stable Coronary Disease. N. Engl. J. Med..

[68] Casado Arroyo R., Polo-Tomas M., Roncalés M.P., Scheiman J., Lanas A. (2012). Lower GI bleeding is more common than upper among patients on dual antiplatelet therapy: Long-term follow-up of a cohort of patients commonly using PPI co-therapy. Heart.

[69] Lai K.C., Lam S.K., Chu K.M., Wong B.C., Hui W.M., Hu W.H., Lau G.K., Wong W.M., Yuen M.F., Chan A.O. (2002). Lansoprazole for the prevention of recurrences of ulcer complications from long-term low-dose aspirin use. N. Engl. J. Med..

[70] Lehault W.B., Hughes D.M. (2017). Review of the Long-Term Effects of Proton Pump Inhibitors. Fed. Pract..

[71] Rassen J.A., Choudhry N.K., Avorn J., Schneeweiss S. (2009). Cardiovascular outcomes and mortality in patients using clopidogrel with proton pump inhibitors after percutaneous coronary intervention or acute coronary syndrome. Circulation.

[72] Grundy S.M., Stone N.J., Bailey A.L., Beam C., Birtcher K.K., Blumenthal R.S., Braun L.T., De Ferranti S., Faiella-Tommasino J., Forman D.E. (2019). 2018 AHA/ACC/AACVPR/AAPA/ABC/ACPM/ADA/AGS/APhA/ASPC/NLA/PCNA guideline on the management of blood cholesterol: A report of the American College of Cardiology/American Heart Association Task Force on Clinical Practice Guidelines. Circulation.

[73] Gencer B., Marston N.A., Im K., Cannon C.P., Sever P., Keech A., Braunwald E., Giugliano R.P., Sabatine M.S. (2020). Efficacy and safety of lowering LDL cholesterol in older patients: A systematic review and meta-analysis of randomised controlled trials. Lancet.

[74] Lee S.-H., Lee Y.-J., Heo J.H., Hur S.-H., Choi H.H., Kim K.-J., Kim J.H., Park K.-H., Lee J.H., Choi Y.J. (2023). Combination moderate-intensity statin and ezetimibe therapy for elderly patients with atherosclerosis. J. Am. Coll. Cardiol..

[75] Mach F., Baigent C., Catapano A.L., Koskinas K.C., Casula M., Badimon L., Chapman M.J., De Backer G.G., Delgado V., Ference B.A. (2019). 2019 ESC/EAS Guidelines for the management of dyslipidaemias: Lipid modification to reduce cardiovascular risk. Atherosclerosis.

[76] Densham E., Youssef E., Ferguson O., Winter R. (2024). The effect of statins on falls and physical activity in people aged 65 and older: A systematic review. Eur. J. Clin. Pharmacol..

[77] Zhou Z., Ryan J., Ernst M.E., Zoungas S., Tonkin A.M., Woods R.L., McNeil J.J., Reid C.M., Curtis A.J., Wolfe R. (2021). Effect of statin therapy on cognitive decline and incident dementia in older adults. J. Am. Coll. Cardiol..

[78] Virani S.S., Newby L.K., Arnold S.V., Bittner V., Brewer L.C., Demeter S.H., Dixon D.L., Fearon W.F., Hess B., Johnson H.M. (2023). 2023 AHA/ACC/ACCP/ASPC/NLA/PCNA Guideline for the Management of Patients With Chronic Coronary Disease: A Report of the American Heart Association/American College of Cardiology Joint Committee on Clinical Practice Guidelines. Circulation.

[79] Dahl Aarvik M., Sandven I., Dondo T.B., Gale C.P., Ruddox V., Munkhaugen J., Atar D., Otterstad J.E. (2019). Effect of oral β-blocker treatment on mortality in contemporary post-myocardial infarction patients: A systematic review and meta-analysis. Eur. Heart J. Cardiovasc. Pharmacother..

[80] Bangalore S., Makani H., Radford M., Thakur K., Toklu B., Katz S.D., DiNicolantonio J.J., Devereaux P.J., Alexander K.P., Wetterslev J. (2014). Clinical outcomes with β-blockers for myocardial infarction: A meta-analysis of randomized trials. Am. J. Med..

[81] Korhonen M.J., Robinson J.G., Annis I.E., Hickson R.P., Bell J.S., Hartikainen J., Fang G. (2017). Adherence Tradeoff to Multiple Preventive Therapies and All-Cause Mortality After Acute Myocardial Infarction. J. Am. Coll. Cardiol..

[82] Rossello X., Raposeiras-Roubin S., Latini R., Dominguez-Rodriguez A., Barrabés J.A., Sánchez P.L., Anguita M., Fernández-Vázquez F., Pascual-Figal D., De la Torre Hernandez J.M. (2022). Rationale and design of the pragmatic clinical trial tREatment with Beta-blockers after myOcardial infarction withOut reduced ejection fracTion (REBOOT). Eur. Heart J. Cardiovasc. Pharmacother..

[83] Munkhaugen J., Ruddox V., Halvorsen S., Dammen T., Fagerland M.W., Hernæs K.H., Vethe N.T., Prescott E., Jensen S.E., Rødevand O. (2019). BEtablocker Treatment After acute Myocardial Infarction in revascularized patients without reduced left ventricular ejection fraction (BETAMI): Rationale and design of a prospective, randomized, open, blinded end point study. Am. Heart J..

[84] Kristensen A.M.D., Bovin A., Zwisler A.D., Cerquira C., Torp-Pedersen C., Bøtker H.E., Gustafsson I., Veien K.T., Thomsen K.K., Olsen M.H. (2020). Design and rationale of the Danish trial of beta-blocker treatment after myocardial infarction without reduced ejection fraction: Study protocol for a randomized controlled trial. Trials.

[85] Yndigegn T., Lindahl B., Mars K., Alfredsson J., Benatar J., Brandin L., Erlinge D., Hallen O., Held C., Hjalmarsson P. (2024). Beta-Blockers after Myocardial Infarction and Preserved Ejection Fraction. N. Engl. J. Med..

[86] Liu H.H., Li S., Zhang Y., Zhang M., Zhang H.W., Qian J., Dou K.F., Li J.J. (2024). Association of β-blocker use at discharge and prognosis of oldest old with acute myocardial infarction: A prospective cohort study. Eur. Geriatr. Med..

[87] Zullo A.R., Olean M., Berry S.D., Lee Y., Tjia J., Steinman M.A. (2019). Patient-Important Adverse Events of β-blockers in Frail Older Adults after Acute Myocardial Infarction. J. Gerontol. A Biol. Sci. Med. Sci..

[88] Shavadia J.S., Holmes D.N., Thomas L., Peterson E.D., Granger C.B., Roe M.T., Wang T.Y. (2019). Comparative Effectiveness of β-Blocker Use Beyond 3 Years After Myocardial Infarction and Long-Term Outcomes Among Elderly Patients. Circ. Cardiovasc. Qual. Outcomes.

[89] Ishak D., Aktaa S., Lindhagen L., Alfredsson J., Dondo T.B., Held C., Jernberg T., Yndigegn T., Gale C.P., Batra G. (2023). Association of beta-blockers beyond 1 year after myocardial infarction and cardiovascular outcomes. Heart.

[90] Park C.S., Yang H.-M., Ki Y.-J., Kang J., Han J.-K., Park K.W., Kang H.-J., Koo B.-K., Kim C.-J., Cho M.C. (2021). Left ventricular ejection fraction 1 year after acute myocardial infarction identifies the benefits of the long-term use of β-blockers: Analysis of data from the KAMIR-NIH registry. Circ. Cardiovasc. Interv..

[91] Dondo T.B., Hall M., West R.M., Jernberg T., Lindahl B., Bueno H., Danchin N., Deanfield J.E., Hemingway H., Fox K.A.A. (2017). β-Blockers and Mortality After Acute Myocardial Infarction in Patients Without Heart Failure or Ventricular Dysfunction. J. Am. Coll. Cardiol..

[92] Holt A., Blanche P., Zareini B., Rajan D., El-Sheikh M., Schjerning A.-M., Schou M., Torp-Pedersen C., McGettigan P., Gislason G.H. (2021). Effect of long-term beta-blocker treatment following myocardial infarction among stable, optimally treated patients without heart failure in the reperfusion era: A Danish, nationwide cohort study. Eur. Heart J..

[93] Park J.J., Kim S.H., Kang S.H., Yoon C.H., Cho Y.S., Youn T.J., Chae I.H., Choi D.J. (2018). Effect of β-Blockers Beyond 3 Years After Acute Myocardial Infarction. J. Am. Heart Assoc..

[94] Puymirat E., Riant E., Aissaoui N., Soria A., Ducrocq G., Coste P., Cottin Y., Aupetit J.F., Bonnefoy E., Blanchard D. (2016). β blockers and mortality after myocardial infarction in patients without heart failure: Multicentre prospective cohort study. BMJ.

[95] Neumann A., Maura G., Weill A., Alla F., Danchin N. (2018). Clinical Events After Discontinuation of β-Blockers in Patients Without Heart Failure Optimally Treated After Acute Myocardial Infarction: A Cohort Study on the French Healthcare Databases. Circ. Cardiovasc. Qual. Outcomes.

[96] Kim J., Kang D., Park H., Kang M., Park T.K., Lee J.M., Yang J.H., Song Y.B., Choi J.-H., Choi S.-H. (2020). Long-term β-blocker therapy and clinical outcomes after acute myocardial infarction in patients without heart failure: Nationwide cohort study. Eur. Heart J..

[97] Pfeffer M.A. (1993). The Survival and Ventricular Enlargement (SAVE) study: Rationale and perspective. Herz.

[98] The Acute Infarction Ramipril Efficacy (AIRE) Study Investigators (1993). Effect of ramipril on mortality and morbidity of survivors of acute myocardial infarction with clinical evidence of heart failure. Lancet.

[99] Ahn W.J., Rha S.W., Choi B.G., Jeong M.H. (2023). The impact of angiotensin-converting-enzyme inhibitors versus angiotensin receptor blockers on 3-year clinical outcomes in elderly (≥ 65) patients with acute myocardial infarction without hypertension. Heart Vessels.

[100] Yusuf S., Teo K., Anderson C., Pogue J., Dyal L., Copland I., Schumacher H., Dagenais G., Sleight P. (2008). Effects of the angiotensin-receptor blocker telmisartan on cardiovascular events in high-risk patients intolerant to angiotensin-converting enzyme inhibitors: A randomised controlled trial. Lancet.

[101] Pfeffer M.A., McMurray J.J., Velazquez E.J., Rouleau J.L., Køber L., Maggioni A.P., Solomon S.D., Swedberg K., Van de Werf F., White H. (2003). Valsartan, captopril, or both in myocardial infarction complicated by heart failure, left ventricular dysfunction, or both. N. Engl. J. Med..

[102] Dickstein K., Kjekshus J. (2002). Effects of losartan and captopril on mortality and morbidity in high-risk patients after acute myocardial infarction: The OPTIMAAL randomised trial. Optimal Trial in Myocardial Infarction with Angiotensin II Antagonist Losartan. Lancet.

[103] Pappoe L.S., Winkelmayer W.C. (2010). ACE inhibitor and angiotensin II type 1 receptor antagonist therapies in elderly patients with diabetes mellitus: Are they underutilized?. Drugs Aging.

[104] Fang G., Annis I.E., Farley J.F., Mahendraratnam N., Hickson R.P., Stürmer T., Robinson J.G. (2018). Incidence of and Risk Factors for Severe Adverse Events in Elderly Patients Taking Angiotensin-Converting Enzyme Inhibitors or Angiotensin II Receptor Blockers after an Acute Myocardial Infarction. Pharmacotherapy.

[105] Abdel-Rahman E.M., Alhamad T., Reeves W.B., Awad A.S. (2012). Management of Diabetic Nephropathy in the Elderly: Special Considerations. J. Nephrol. Ther..

[106] Gray S.L., LaCroix A.Z., Aragaki A.K., McDermott M., Cochrane B.B., Kooperberg C.L., Murray A.M., Rodriguez B., Black H., Woods N.F. (2009). Angiotensin-converting enzyme inhibitor use and incident frailty in women aged 65 and older: Prospective findings from the Women’s Health Initiative Observational Study. J. Am. Geriatr. Soc..

[107] Sumukadas D., Witham M.D., Struthers A.D., McMurdo M.E. (2007). Effect of perindopril on physical function in elderly people with functional impairment: A randomized controlled trial. CMAJ.

[108] Ho J.K., Moriarty F., Manly J.J., Larson E.B., Evans D.A., Rajan K.B., Hudak E.M., Hassan L., Liu E., Sato N. (2021). Blood-Brain Barrier Crossing Renin-Angiotensin Drugs and Cognition in the Elderly: A Meta-Analysis. Hypertension.

[109] Goel S.S., Kleiman N.S., Zoghbi W.A., Reardon M.J., Kapadia S.R. (2020). Renin-Angiotensin System Blockade in Aortic Stenosis: Implications Before and After Aortic Valve Replacement. J. Am. Heart Assoc..

[110] Pitt B., Remme W., Zannad F., Neaton J., Martinez F., Roniker B., Bittman R., Hurley S., Kleiman J., Gatlin M. (2003). Eplerenone, a Selective Aldosterone Blocker, in Patients with Left Ventricular Dysfunction after Myocardial Infarction. N. Engl. J. Med..

[111] Pfeffer M.A., Claggett B., Lewis E.F., Granger C.B., Køber L., Maggioni A.P., Mann D.L., McMurray J.J.V., Rouleau J.L., Solomon S.D. (2021). Angiotensin Receptor-Neprilysin Inhibition in Acute Myocardial Infarction. N. Engl. J. Med..

[112] Kwon O., Myong J.P., Lee Y., Choi Y.J., Yi J.E., Seo S.M., Jang S.W., Kim P.J., Lee J.M. (2023). Sodium-Glucose Cotransporter-2 Inhibitors After Acute Myocardial Infarction in Patients With Type 2 Diabetes: A Population-Based Investigation. J. Am. Heart Assoc..

[113] Moady G., Yakubovich I., Atar S. (2024). Safety and Efficacy of Early SGLT2 Inhibitors Initiation in Diabetic Patients Following Acute Myocardial Infarction, a Retrospective Study. J. Cardiovasc. Pharmacol. Ther..

[114] Goldberg R.J., Steg P.G., Sadiq I., Granger C.B., Jackson E.A., Budaj A., Brieger D., Avezum A., Goodman S. (2002). Extent of, and factors associated with, delay to hospital presentation in patients with acute coronary disease (the GRACE registry). Am. J. Cardiol..

[115] Alexander K.P., Newby L.K., Armstrong P.W., Cannon C.P., Gibler W.B., Rich M.W., Van de Werf F., White H.D., Weaver W.D., Naylor M.D. (2007). Acute coronary care in the elderly, part II: ST-segment-elevation myocardial infarction: A scientific statement for healthcare professionals from the American Heart Association Council on Clinical Cardiology: In collaboration with the Society of Geriatric Cardiology. Circulation.

[116] Bueno H., Betriu A., Heras M., Alonso J.J., Cequier A., García E.J., López-Sendón J.L., Macaya C., Hernández-Antolín R. (2011). Primary angioplasty vs. fibrinolysis in very old patients with acute myocardial infarction: TRIANA (TRatamiento del Infarto Agudo de miocardio eN Ancianos) randomized trial and pooled analysis with previous studies. Eur. Heart J..

[117] de Boer S.P., Westerhout C.M., Simes R.J., Granger C.B., Zijlstra F., Boersma E. (2010). Mortality and morbidity reduction by primary percutaneous coronary intervention is independent of the patient’s age. JACC Cardiovasc. Interv..

[118] Fox K.A., Dabbous O.H., Goldberg R.J., Pieper K.S., Eagle K.A., Van de Werf F., Avezum A., Goodman S.G., Flather M.D., Anderson F.A. (2006). Prediction of risk of death and myocardial infarction in the six months after presentation with acute coronary syndrome: Prospective multinational observational study (GRACE). BMJ.

[119] Antman E.M., Cohen M., Bernink P.J., McCabe C.H., Horacek T., Papuchis G., Mautner B., Corbalan R., Radley D., Braunwald E. (2000). The TIMI risk score for unstable angina/non-ST elevation MI: A method for prognostication and therapeutic decision making. JAMA.

[120] Gómez-Talavera S., Núñez-Gil I., Vivas D., Ruiz-Mateos B., Viana-Tejedor A., Martín-García A., Higueras-Nafría J., Macaya C., Fernández-Ortiz A. (2014). [Acute coronary syndrome in nonagenarians: Clinical evolution and validation of the main risk scores]. Rev. Esp. Geriatr. Gerontol..

[121] Alexander K.P., Newby L.K., Cannon C.P., Armstrong P.W., Gibler W.B., Rich M.W., Van de Werf F., White H.D., Weaver W.D., Naylor M.D. (2007). Acute coronary care in the elderly, part I: Non-ST-segment-elevation acute coronary syndromes: A scientific statement for healthcare professionals from the American Heart Association Council on Clinical Cardiology: In collaboration with the Society of Geriatric Cardiology. Circulation.

[122] Zhu X.Y., Zhang K.J., Li X., Su F.F., Tian J.W. (2024). Prognostic value of Geriatric Nutritional Risk Index and systemic immune-inflammatory index in elderly patients with acute coronary syndromes. Sci. Rep..

[123] Kunniardy P., Koshy A.N., Meehan G., Murphy A.C., Ramchand J., Clark D.J., Farouque O., Yudi M.B. (2022). Invasive versus conservative management in patients aged ≥85 years presenting with non-ST-elevation myocardial infarction. Intern. Med. J..

[124] Sanchis J., Bueno H., Miñana G., Guerrero C., Martí D., Martínez-Sellés M., Domínguez-Pérez L., Díez-Villanueva P., Barrabés J.A., Marín F. (2023). Effect of Routine Invasive vs Conservative Strategy in Older Adults With Frailty and Non–ST-Segment Elevation Acute Myocardial Infarction: A Randomized Clinical Trial. JAMA Intern. Med..

[125] Reaño J.D.P., Shiu L.A.B., Miralles K.V., Dimalala M.G.C., Pestaño N.S., Punzalan F.E.R., Tumanan-Mendoza B., Reyes M.J.T., Castillo R.R. (2020). A systematic review and meta-analysis on the effectiveness of an invasive strategy compared to a conservative approach in patients > 65 years old with non-ST elevation acute coronary syndrome. PLoS ONE.

[126] Ma W., Liang Y., Zhu J. (2018). Early Invasive Versus Initially Conservative Strategy in Elderly Patients Older Than 75 Years with Non-ST-Elevation Acute Coronary Syndrome: A Meta-Analysis. Heart Lung Circ..

[127] Morici N., De Servi S., Toso A., Murena E., Piscione F., Bolognese L., Petronio A.S., Antonicelli R., Cavallini C., Angeli F. (2015). Renal dysfunction, coronary revascularization and mortality among elderly patients with non ST elevation acute coronary syndrome. Eur. Heart J. Acute Cardiovasc. Care.

[128] Go A.S., Chertow G.M., Fan D., McCulloch C.E., Hsu C.Y. (2004). Chronic kidney disease and the risks of death, cardiovascular events, and hospitalization. N. Engl. J. Med..

[129] Fox C.S., Muntner P., Chen A.Y., Alexander K.P., Roe M.T., Cannon C.P., Saucedo J.F., Kontos M.C., Wiviott S.D. (2010). Use of evidence-based therapies in short-term outcomes of ST-segment elevation myocardial infarction and non-ST-segment elevation myocardial infarction in patients with chronic kidney disease: A report from the National Cardiovascular Data Acute Coronary Treatment and Intervention Outcomes Network registry. Circulation.

[130] De Rosa R., Morici N., De Servi S., De Luca G., Galasso G., Piscione F., Ferri L.A., Piatti L., Grosseto D., Tortorella G. (2020). Impact of renal dysfunction and acute kidney injury on outcome in elderly patients with acute coronary syndrome undergoing percutaneous coronary intervention. Eur. Heart J. Acute Cardiovasc. Care.

[131] Bromage D.I., Jones D.A., Rathod K.S., Grout C., Iqbal M.B., Lim P., Jain A., Kalra S.S., Crake T., Astroulakis Z. (2016). Outcome of 1051 Octogenarian Patients With ST-Segment Elevation Myocardial Infarction Treated With Primary Percutaneous Coronary Intervention: Observational Cohort From the London Heart Attack Group. J. Am. Heart Assoc..

[132] Böhm F., Mogensen B., Engstrøm T., Stankovic G., Srdanovic I., Lønborg J., Zwackman S., Hamid M., Kellerth T., Lauermann J. (2024). FFR-Guided Complete or Culprit-Only PCI in Patients with Myocardial Infarction. N. Engl. J. Med..

[133] Joshi F.R., Lønborg J., Sadjadieh G., Helqvist S., Holmvang L., Sørensen R., Jørgensen E., Pedersen F., Tilsted H.H., Høfsten D. (2021). The benefit of complete revascularization after primary PCI for STEMI is attenuated by increasing age: Results from the DANAMI-3-PRIMULTI randomized study. Catheter. Cardiovasc. Interv..

[134] Biscaglia S., Guiducci V., Escaned J., Moreno R., Lanzilotti V., Santarelli A., Cerrato E., Sacchetta G., Jurado-Roman A., Menozzi A. (2023). Complete or Culprit-Only PCI in Older Patients with Myocardial Infarction. N. Engl. J. Med..

[135] Numasawa Y., Inohara T., Ishii H., Yamaji K., Kohsaka S., Sawano M., Kodaira M., Uemura S., Kadota K., Amano T. (2019). Comparison of Outcomes After Percutaneous Coronary Intervention in Elderly Patients, Including 10 628 Nonagenarians: Insights From a Japanese Nationwide Registry (J-PCI Registry). J. Am. Heart Assoc..

[136] De Luca L., Olivari Z., Farina A., Gonzini L., Lucci D., Di Chiara A., Casella G., Chiarella F., Boccanelli A., Di Pasquale G. (2015). Temporal trends in the epidemiology, management, and outcome of patients with cardiogenic shock complicating acute coronary syndromes. Eur. J. Heart Fail..

[137] Lim H.S., Farouque O., Andrianopoulos N., Yan B.P., Lim C.C., Brennan A.L., Reid C.M., Freeman M., Charter K., Black A. (2009). Survival of elderly patients undergoing percutaneous coronary intervention for acute myocardial infarction complicated by cardiogenic shock. JACC Cardiovasc. Interv..

[138] Lim H.S., Andrianopoulos N., Sugumar H., Stub D., Brennan A.L., Lim C.C., Barlis P., Van Gaal W., Reid C.M., Charter K. (2015). Long-term survival of elderly patients undergoing percutaneous coronary intervention for myocardial infarction complicated by cardiogenic shock. Int. J. Cardiol..

[139] Blumer V., Kanwar M.K., Barnett C.F., Cowger J.A., Damluji A.A., Farr M., Goodlin S.J., Katz J.N., McIlvennan C.K., Sinha S.S. (2024). Cardiogenic Shock in Older Adults: A Focus on Age-Associated Risks and Approach to Management: A Scientific Statement From the American Heart Association. Circulation.

[140] Shah A.I., Alabaster A., Dontsi M., Rana J.S., Solomon M.D., Krishnaswami A. (2022). Comparison of coronary revascularization strategies in older adults presenting with acute coronary syndromes. J. Am. Geriatr. Soc..

[141] Gimbel M.E., Willemsen L.M., Daggelders M.C., Kelder J.C., Oirbans T., Beukema K.F., Daeter E.J., Ten Berg J.M. (2020). Long-term follow-up after bypass surgery or coronary stenting in elderly with multivessel disease. Neth. Heart J..

[142] Lemaire A., Soto C., Salgueiro L., Ikegami H., Russo M.J., Lee L.Y. (2020). The impact of age on outcomes of coronary artery bypass grafting. J. Cardiothorac. Surg..

[143] Cacciatore S., Spadafora L., Bernardi M., Galli M., Betti M., Perone F., Nicolaio G., Marzetti E., Martone A.M., Landi F. (2023). Management of Coronary Artery Disease in Older Adults: Recent Advances and Gaps in Evidence. J. Clin. Med..

[144] Gudnadottir G.S., Gudnason T., Wilhelmson K., Ravn-Fischer A. (2022). Multimorbidity and Readmissions in Older People with Acute Coronary Syndromes. Cardiology.

[145] O’Neill D., Forman D.E. (2019). Never Too Old for Cardiac Rehabilitation. Clin. Geriatr. Med..

[146] Suaya J.A., Stason W.B., Ades P.A., Normand S.-L.T., Shepard D.S. (2009). Cardiac Rehabilitation and Survival in Older Coronary Patients. J. Am. Coll. Cardiol..

[147] Ritchey M.D., Maresh S., McNeely J., Shaffer T., Jackson S.L., Keteyian S.J., Brawner C.A., Whooley M.A., Chang T., Stolp H. (2020). Tracking Cardiac Rehabilitation Participation and Completion Among Medicare Beneficiaries to Inform the Efforts of a National Initiative. Circ. Cardiovasc. Qual. Outcomes.

[148] Hammill B.G., Curtis L.H., Schulman K.A., Whellan D.J. (2010). Relationship between cardiac rehabilitation and long-term risks of death and myocardial infarction among elderly Medicare beneficiaries. Circulation.

[149] Inouye S.K., Westendorp R.G., Saczynski J.S. (2014). Delirium in elderly people. Lancet.

[150] Noriega F.J., Vidán M.T., Sánchez E., Díaz A., Serra-Rexach J.A., Fernández-Avilés F., Bueno H. (2015). Incidence and impact of delirium on clinical and functional outcomes in older patients hospitalized for acute cardiac diseases. Am. Heart J..

[151] Tonet E., Campo G., Maietti E., Formiga F., Martinez-Sellés M., Pavasini R., Biscaglia S., Serenelli M., Sanchis J., Diez-Villanueva P. (2020). Nutritional status and all-cause mortality in older adults with acute coronary syndrome. Clin. Nutr..

[152] Ho P.M., Bryson C.L., Rumsfeld J.S. (2009). Medication adherence: Its importance in cardiovascular outcomes. Circulation.

[153] Halvorsen S., Mehilli J., Cassese S., Hall T.S., Abdelhamid M., Barbato E., De Hert S., de Laval I., Geisler T., Hinterbuchner L. (2022). 2022 ESC Guidelines on cardiovascular assessment and management of patients undergoing non-cardiac surgery. Eur. Heart J..

[154] Smilowitz N.R., Berger J.S. (2020). Perioperative cardiovascular risk assessment and management for noncardiac surgery: A review. JAMA.

[155] Rostagno C., Cartei A., Rubbieri G., Ceccofiglio A., Magni A., Forni S., Civinini R., Boccaccini A. (2020). Perioperative Myocardial Infarction/Myocardial Injury Is Associated with High Hospital Mortality in Elderly Patients Undergoing Hip Fracture Surgery. J. Clin. Med..

[156] Domienik-Karłowicz J., Kupczyńska K., Michalski B., Kapłon-Cieślicka A., Darocha S., Dobrowolski P., Wybraniec M., Wańha W., Jaguszewski M. (2021). Fourth universal definition of myocardial infarction. Selected messages from the European Society of Cardiology document and lessons learned from the new guidelines on ST-segment elevation myocardial infarction and non-ST-segment elevation-acute coronary syndrome. Cardiol. J..

[157] Kashlan B., Kinno M., Syed M. (2024). Perioperative myocardial injury and infarction after noncardiac surgery: A review of pathophysiology, diagnosis, and management. Front. Cardiovasc. Med..

[158] Rostagno C., Craighero A. (2024). Postoperative Myocardial Infarction after Non-Cardiac Surgery: An Update. J. Clin. Med..

[159] Puelacher C., Lurati Buse G., Seeberger D., Sazgary L., Marbot S., Lampart A., Espinola J., Kindler C., Hammerer A., Seeberger E. (2018). Perioperative Myocardial Injury After Noncardiac Surgery: Incidence, Mortality, and Characterization. Circulation.

[160] Fabbian F., De Giorgi A., Pala M., Tiseo R., Portaluppi F. (2011). Elevated NT-proBNP levels should be interpreted in elderly patients presenting with dyspnea. Eur. J. Intern. Med..

[161] Gao L., Chen L., He J., Wang B., Liu C., Wang R., Fan L., Cheng R. (2022). Perioperative Myocardial Injury/Infarction After Non-cardiac Surgery in Elderly Patients. Front. Cardiovasc. Med..

[162] Devereaux P.J., Szczeklik W. (2020). Myocardial injury after non-cardiac surgery: Diagnosis and management. Eur. Heart J..

[163] Eagle K.A., Berger P.B., Calkins H., Chaitman B.R., Ewy G.A., Fleischmann K.E., Fleisher L.A., Froehlich J.B., Gusberg R.J., Leppo J.A. (2002). ACC/AHA guideline update for perioperative cardiovascular evaluation for noncardiac surgery—Executive summary a report of the American College of Cardiology/American Heart Association Task Force on Practice Guidelines (Committee to Update the 1996 Guidelines on Perioperative Cardiovascular Evaluation for Noncardiac Surgery). Circulation.

[164] Graham M.M., Sessler D.I., Parlow J.L., Biccard B.M., Guyatt G., Leslie K., Chan M.T.V., Meyhoff C.S., Xavier D., Sigamani A. (2018). Aspirin in Patients With Previous Percutaneous Coronary Intervention Undergoing Noncardiac Surgery. Ann. Intern. Med..

